# Mililatensols A–C, New Records of Sarsolenane and Capnosane Diterpenes from Soft Coral *Sarcophyton mililatensis*

**DOI:** 10.3390/md20090566

**Published:** 2022-09-06

**Authors:** Qing Bu, Min Yang, Xian-Yun Yan, Song-Wei Li, Zeng-Yue Ge, Ling Zhang, Li-Gong Yao, Yue-Wei Guo, Lin-Fu Liang

**Affiliations:** 1 College of Materials Science and Engineering, Central South University of Forestry and Technology, 498 South Shaoshan Road, Changsha 410004, China; 2 State Key Laboratory of Drug Research, Shanghai Institute of Materia Medica, Chinese Academy of Sciences, 555 Zu Chong Zhi Road, Zhangjiang Hi-Tech Park, Shanghai 201203, China; 3 Shandong Laboratory of Yantai Drug Discovery, Bohai Rim Advanced Research Institute for Drug Discovery, Yantai 264117, China; 4 Open Studio for Druggability Research of Marine Natural Products, Pilot National Laboratory for Marine Science and Technology (Qingdao), 1 Wenhai Road, Aoshanwei, Jimo, Qingdao 266237, China; 5 Collaborative Innovation Center of Yangtze River Delta Region Green Pharmaceuticals and College of Pharmaceutical Science, Zhejiang University of Technology, Hangzhou 310014, China

**Keywords:** soft coral, *Sarcophyton mililatensis*, sarsolenane, capnosane, stereochemistry, anti-inflammatory activity

## Abstract

Three unusual diterpenes with rare sarsolenane and capnosane skeletons, namely mililatensols A–C (**1**–**3**), were isolated from the South China Sea soft coral *Sarcophyton mililatensis*, leading to the first record of sarsolenane and capnosane diterpenes from the title animal. The structures of compounds **1**–**3** were established by extensive spectroscopic analysis and comparison with the literature data. Moreover, the absolute configuration of **2** was determined by TDDFT ECD calculations. In an in vitro bioassay, none of the isolated compounds showed obvious anti-inflammatory activity on LPS-induced TNF-*α* release in RAW264.7 macrophages. In the preliminary virtual screening of inhibitory potential against SARS-CoV-2 by molecular docking, the results showed these three diterpenes were potential SARS-CoV-2 M^pro^ inhibitors.

## 1. Introduction

Literature survey focusing on the secondary metabolites of soft corals of the genus *Sarcophyton* revealed that they are a reservoir of diverse natural products, particularly diterpenes. Till now, more than 300 diterpenes have been reported from ca. 18 species of this genus, except undefined species. Moreover, many diterpenes exhibited a broad range of notable biological activities, such as anti-inflammatory effects [1,2]. Their intriguing scaffolds and excellent bioactivities have gained great attention from synthetic chemists as challenging targets for total synthesis [3,4].

Of them, sarsolenane diterpenes are extremely rare in nature, represented so far by just five compounds from the above-mentioned genus. They are sarsolenone and 7-deacetyl-sarsolenone from the soft coral *Sarcophyton solidum* [5,6], and dihydrosarsolenone, methyl dihydrosarsolenoneate, and secodihydrosarsolenone from the soft coral *Sarcophyton trocheliophorum* [7,8]. The absolute configuration of dihydrosarsolenone was established by TDDFT ECD calculations, while the absolute configurations of methyl dihydrosarsolenoneate and 7-deacetyl-sarsolenone were deduced from the comparison of their ECD spectra with those of the corresponding reference compounds, respectively [6,7]. To date, dihydrosarsolenone, methyl dihydrosarsolenoneate, and secodihydrosarsolenone have merely been subjected to the bioassay, and the results showed that only secodihydrosarsolenone exhibited moderate PTP1B inhibitory activity [7,8].

Capnosane diterpenes are also in the minority of diterpenes from the genus *Sarcophyton*, which include sarsolilides A–C from the soft corals *S. solidum* [6,9] and *S. trocheliophorum* [7], sarcophyolides B–D from the soft coral *Sarcophyton elegans* [10], and trocheliophols A–S and sarcophytrols A–C from the soft coral *S. trocheliophorum* [11,12]. To establish the absolute configurations of capnosane diterpenes, different techniques, such as TDDFT ECD calculations, X-ray diffraction, the modified Mosher’s method, and CD were applied [7,10,11,12]. Only the inhibitory effects of capnosane diterpenes against PTP1B, inflammation-related NF-κB, bacterial pathogens, and tumor cell lines were evaluated. The results showed that sarsolilides A and B were potential PTP1B inhibitors [7] and sarcophyolide B was cytotoxic against A2780 human ovarian tumor cells [10], while the trocheliophols H, I, and S showed inhibitory potential against phytopathogens and human disease-related Gram-positive and Gram-negative bacteria [11]. Interestingly, two cembrane–capnosane heterodimers, bissubvilides A and B, and sarsolilide B were discovered in the soft coral *Sarcophyton subviride* recently [13].

**Figure 1:**
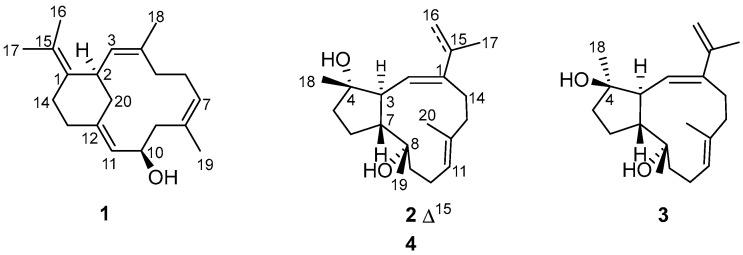
Structures of compounds **1**–**4**.

In the course of our ongoing research aiming for structurally novel and biologically active secondary metabolites from South China Sea soft corals [14,15,16,17,18,19], the title animal *Sarcophyton mililatensis* was collected from the Xigu Island, Hainan Province, China. Among all of the *Sarcophyton* species, *S. mililatensis* has rarely been investigated. There are only four reports of the chemical constituents and bioactivities of *S. mililatensis* [18,20,21,22]. Our previous chemical investigation on the South China Sea soft coral *S. mililatensis* led to the discovery of sarcomililate A, an unprecedented diterpenoid with a tricyclo [11.3.0.0^2,16^]hexadecane carbon framework [18]. Inspired by this research, and in order to disclose more chemically intriguing marine natural products, especially trace constituents, we recently conducted a continuing chemical investigation of the Et_2_O-soluble extract of the title soft coral. This study resulted in the isolation and characterization of three unusual diterpenes mililatensols A–C (**1**–**3**) bearing the rare sarsolenane and capnosane carbon frameworks (Figure 1). Hereto, the isolation, structure elucidation, anti-inflammatory, and SARS-CoV-2 M^pro^-inhibitory activities of these new isolates are described.

## 2. Results and Discussion

The acetone extract of the soft coral *S. mililatensis* was partitioned between Et_2_O and H_2_O. The Et_2_O-soluble portion was repeatedly chromatographed over silica gel, Sephadex LH-20, and RP-HPLC to yield the new records of sarsolenane and capnosane diterpenes, namely mililatensols A–C (**1**–**3**) (Figure 1).

**Figure 2:**
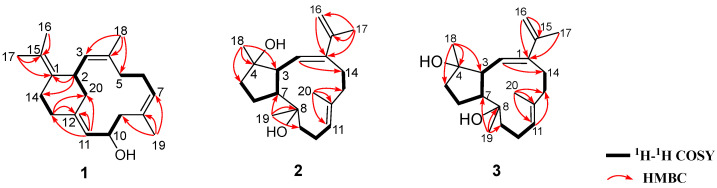
Selected key COSY and HMBC correlations for compounds **1**–**3**.

**Figure 3:**
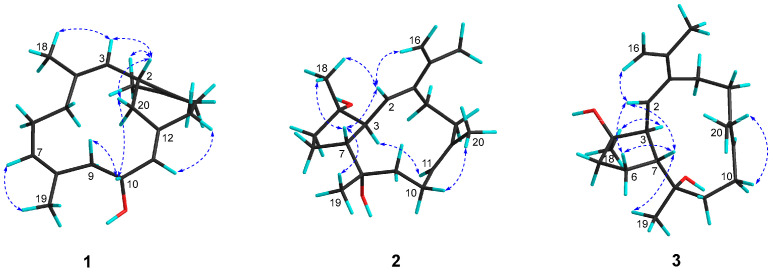
Selected key ROESY correlations for compounds **1**–**3**.

Compound **1**, a white powder, possessed the molecular formula C_20_H_30_O, which was deduced from the molecular ion peak at *m*/*z* 286.2293 ([M]^+^, calcd. for C_20_H_30_O, 286.2291) (Figure S1), indicating six degrees of unsaturation. The IR absorption at 3282 cm^−^^1^ (Figure S8) revealed the existence of the hydroxyl group, which was in agreement with the presence of a secondary alcohol functionality [*δ*_H_ 4.37 (1H, dt, *J* = 2.8, 9.4 Hz, H-10), *δ*_C_ 68.4 (CH, C-10)] as indicated by the ^1^H and ^13^C NMR data (Table 1, Figures S2 and S3). Its ^13^C NMR spectrum exhibited 20 carbon resonances assigned to four methyls, six methylenes, five methines (including three olefinic, *δ*_C_ 126.8, 127.6 and 128.1, and an oxygenated, *δ*_C_ 68.4), and five quaternary carbons (all olefinic, *δ*_C_ 122.6, 130.0, 131.3, 132.5, and 139.4) (Table 1), which were deduced from DEPT and HSQC experiments (Figures S3 and S4). In addition, its ^1^H NMR spectrum (Figure S2) displayed signals of four vinyl methyls at *δ*_H_ 1.64 (3H, s, H_3_-18), 1.64 (3H, s, H_3_-19), 1.67 (3H, s, H_3_-16) and 1.69 (3H, s, H_3_-17), and three olefinic protons at *δ*_H_ 4.95 (1H, t, *J* = 7.4 Hz, H-7), 5.03 (1H, d, *J* = 10.2 Hz, H-3) and 5.20 (1H, d, *J* = 9.4 Hz, H-11), which were attributable to three trisubstituted double bonds (Table 1). As revealed by the ^1^H and ^13^C NMR data, there were four double bonds, accounting for four degrees of unsaturation. The remaining two degrees of unsaturation indicated the presence of two rings in the molecule. Analysis of the ^1^H–^1^H COSY correlations (Figure 2 and Figure S5) readily disclosed four spin-coupling segments from H_2_-5 via H_2_-6 to H-7, from H_2_-9 via H-10 to H-11, from H_2_-13 to H_2_-14, and from H_2_-20 via H-2 to H-3. On the basis of an HMBC experiment (Figure 2 and Figure S6), these four fragments could be fully connected by inserting the “loose ends” of the quaternary carbon atoms of C-1, C-4, C-8, and C-12. The cyclododecane ring was constructed by the characteristic HMBC cross-peaks of H_3_-18/C-3, C-4 and C-5, H_3_-19/C-7, C-8 and C-9, H-11/C-12 and C-13. Moreover, the key HMBC correlations of H-2/C-1 and C-14, H_2_-13/C-12, and C-20 disclosed a typical cyclohexane ring. The joints of the two-carbon framework were C-2 and C-12, also indicated by the above-mentioned HMBC correlations. Furthermore, the HMBC correlations from H_3_-16 to C-15 and from H_3_-17 to C-1 and C-15 revealed a tetrasubstituted double bond Δ^1(15)^ at C-1 in the molecule. Thus, the planar structure of **1** was delineated as a new record of sarsolenane diterpenes. The NOESY (Figure 3 and Figure S7) cross-peaks of H-3/H_3_-18, H-7/H_3_-19, and H-11/H-13a (*δ*_H_ 2.08) indicated that the double bonds Δ^3^, Δ^7^, and Δ^11^ in **1** took *Z*-geometry, respectively. The relative configurations of the two chiral carbons (C-2 and C-10) could be deduced from the NOESY cross-peaks of H-10 (*δ*_H_ 4.37)/H-20a (*δ*_H_ 2.30) and H-2 (*δ*_H_ 3.73)/H-20a. Hereto, the structure of **1** was temporally depicted as shown in Figure 1.

**Figure 4:**
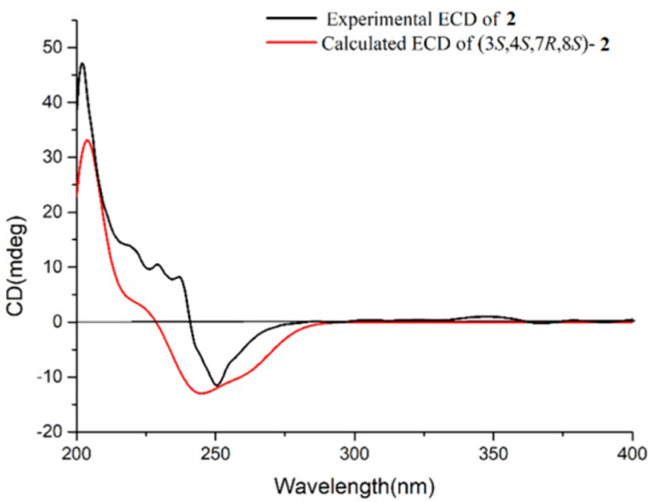
Comparison of experimental ECD spectrum (black) of **2** and predicted ECD spectrum (red) for 3*S*,4*S*,7*R*,8*S*−**2** by TDDFT calculation at the B3LYP/6-311G(d) level.

Compound **2** was obtained as colorless oil. Its molecular formula was ascertained as C_20_H_32_O_2_ by the pseudo-molecular ion peak at *m*/*z* 327.2297 ([M + Na]^+^, calcd. for C_20_H_32_O_2_Na, 327.2295) (Figure S9), requiring five degrees of unsaturation. The IR spectrum of compound **2** (Figure S16) revealed characteristic absorption for hydroxyl at 3445 cm^−1^. Its ^1^H and ^13^C NMR data (Table 1, Figures S10–S14) disclosed the presence of two trisubstituted double bonds (*δ*_H_ 5.54 (1H, d, *J* = 9.6 Hz, H-2), *δ*_C_ 130.0 (C-2), 142.8 (C-1) and *δ*_H_ 5.29 (1H, dd, *J* = 5.4, 10.0 Hz, H-11), *δ*_C_ 127.9 (C-11), 135.4 (C-12)), one terminal double bond (*δ*_H_ 4.99 (1H, s, H-16a), 5.09 (1H, s, H-16b), *δ*_C_ 112.8 (C-16), 141.3 (C-15)), two unprotonated oxygenated carbons (*δ*_C_ 74.8 (C-8), 82.2 (C-4)), two vinyl methyls (*δ*_H_ 1.63 (3H, s, H_3_-20), *δ*_C_ 18.8 (C-20), and *δ*_H_ 1.91 (3H, s, H_3_-17), *δ*_C_ 21.9 (C-17)), and two methyls bonded to tertiary carbons (*δ*_H_ 1.12 (3H, s, H_3_-18), *δ*_C_ 24.2 (C-18), and 1.16 (3H, d, H_3_-19), *δ*_C_ 31.9 (C-19)), accounting for three degrees of unsaturation. The remaining two degrees of unsaturation suggested that **2** was a bicyclic diterpene. Comparison of these spectroscopic data with those of the known compound pavidolide D (**4**) previously isolated from the soft coral *Sinularia pavida* [23] suggested a structural resemblance between them. In fact, a similar substructure of 5/9-fused bicyclo rings was present in both two compounds, which is a typical feature of the capnosane skeleton. The major difference between compounds **2** and **4** centered on the C-1 isopropyl substituent, where the two doublet methyl resonances in **4** were replaced by a pair of singlets of an exo-methylene group (*δ*_H_ 4.99, 5.09; *δ*_C_ 112.8, 141.3) and an allylic singlet methyl (*δ*_H_ 1.91, *δ*_C_ 21.9) in **2** (Figure 1). Interpretation of the diagnostic HMBC correlations from H_3_-17 to C-1, C-15, and C-16 (Figure 2 and Figure S14) associated with the extra one degree of unsaturation in **2** were all consistent with an isopropenyl substituent at C-1. Therefore, compound **2** was concluded to be the 16,17-dehydro derivative of **4**. The (*E*,*E*) geometry of the double bonds Δ^1^ and Δ^11^ in **2** was confirmed upon the observation of the NOESY cross-peaks of H-16b (*δ*_H_ 5.09)/H-2 (*δ*_H_ 5.54) and H-10a (*δ*_H_ 2.34)/H_3_-20 (*δ*_H_ 1.63) (Figure 3 and Figure S15). Furthermore, the NOE interactions of H-7 (*δ*_H_ 2.05)/H_3_-18 (*δ*_H_ 1.12) and H-7/H_3_-19 (*δ*_H_ 1.16), and the lack of H-7/H-3 (*δ*_H_ 2.73) revealed H-7, H_3_-18, and H_3_-19 were co-facial, while H-3 was the opposite. To establish the absolute configuration of **2**, ECD spectra of both enantiomeric forms (3*S*,4*S*,7*R*,8*S* and 3*R*,4*R*,7*S*,8*R*) were calculated by employing time-dependent density functional theory (TDDFT), using NMR-demonstrated conformation (Figure S25) as the initial structure input. The ECD calculations were conducted with the B3LYP/6-311G(d) basis set using the IEFPCM solvent continuum model with CH_3_OH as the solvent. As shown in Figure 4, the experimental ECD spectrum of **2** in CH_3_OH displayed one negative π−π* Cotton effect at 250 nm (Δ*ε* −1.11). Perfect agreement between the experimental ECD spectrum and the calculated one allowed the assignment of the absolute configuration as (3*S*,4*S*,7*R*,8*S*).

The colorless oil **3** had the same molecular formula C_20_H_32_O_2_ as **2**, which was disclosed by the pseudo-molecular ion peak in the HRESIMS experiment (*m*/*z* 327.2298 [M + Na]^+^, calcd. for C_20_H_32_O_2_Na, 327.2295) (Figure S17). The ^1^H and ^13^C NMR (Table 1, Figures S18 and S19) as well as IR data (Figure S24) of **3** closely resembled those of **2**, while 2D NMR (^1^H–^1^H COSY, HSQC, HMBC, Figures S20–S22) analysis revealed both **3** and **2** having the same gross structure (Figure 2). The difference was due to the apparent upfield-shifted H-3 (*δ*_H_ 2.37) in **3** comparison with that (*δ*_H_ 2.73) of **2** accompanied by the downfield-shifted C-18 (*δ*_C_ 26.8), suggesting **3** to be a C-4 epimer of **2** [23]. The presence of NOESY correlation between H-3 and H_3_-18 (*δ*_H_ 1.22) and the lack of NOE interaction between H-7 (*δ*_H_ 2.50) and H_3_-18, as revealed in the ROESY spectrum (Figure 3 and Figure S23) of **3,** further supported the structural assignment. Thus, the absolute configuration of **3** could be assigned as (3*S*,4*R*,7*R*,8*S*).

The discovery of mililatensols A–C (**1**–**3**) with two different carbobicyclic skeletons represents an example of the productivity of the soft coral *S. mililatensis*. It is worth pointing out that this is the first report of sarsolenane and capnosane diterpenes from the title animal. This study, as well as our previous research on the South China Sea soft coral *S. mililatensis,* permitted an upgrade of our knowledge on the structurally diverse marine diterpenes, especially those produced by soft corals of the genus *Sarcophyton*. Moreover, it is intriguing to note that till now, the sarsolenane and capnosane diterpenes have only been co-isolated from two species *S. solidum* [5,6,9] and *S. trocheliophorum* [7] besides *S. mililatensis*. These new findings, as well as the limited previous investigations on the title animal, revealed the rarely studied soft coral *S. mililatensis* is a biochemical warehouse for terpenes.

All the isolates were subjected to the bioassay for anti-inflammatory effects on LPS-induced TNF-*α* release in RAW264.7 macrophages. The results showed that compounds **1**–**3** exhibited the inhibition ratios of 26.8%, 11.4%, and 20.1% at a concentration of 20 μmol/L, indicating none of them possessed obvious activities.

**Figure 5:**
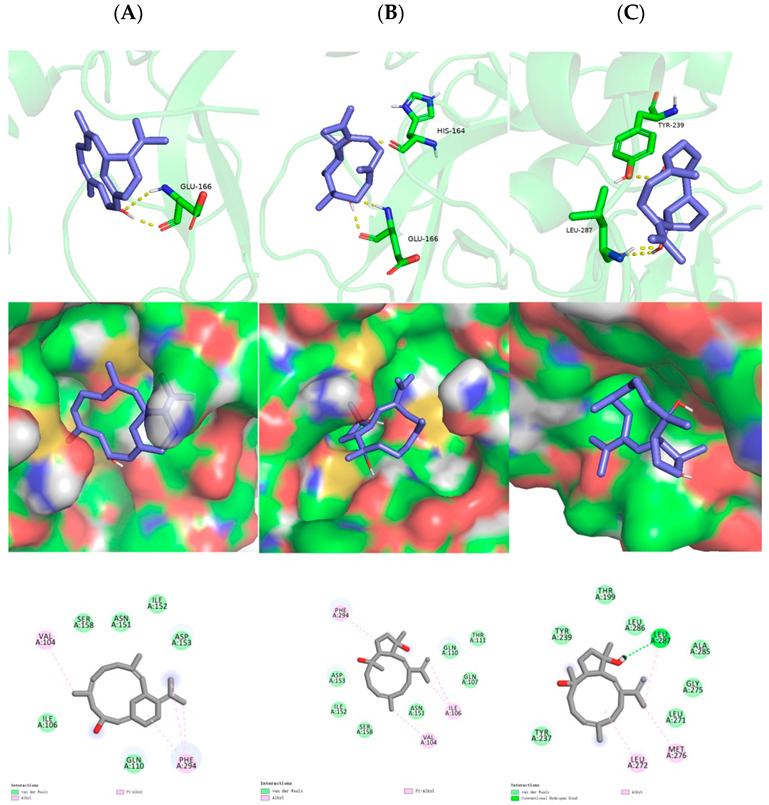
In silico binding modes of compounds **1**–**3** at SARS-CoV-2 M^pro^ crystal structure 6LU7: upper row—the clear combination of hydrogen bonds within the target pocket; middle row—surfaces of 6LU7 with combined compounds; lower row—two-dimensional ligand interaction diagrams of three compounds at the SARS-CoV-2 M^pro^ domain. Left list (**A**) represents docking results of **1**; middle list (**B**) represents docking results of **2**; right list (**C**) represents docking results of **3**.

The preliminary virtual screening for inhibitory potential against SARS-CoV-2 was performed by molecular docking experiments, using the highly resolved SARS-CoV-2 M^pro^ crystal structure (PDB: 6LU7 with a resolution of 2.16 Å). As shown, the hydrogen bond was formed between the C-10 hydroxyl of sarsolenane diterpene **1** and Glu166 (Figure 5A, upper row), which were lying in the active site. Moreover, **1** occupied the hydrophobic pocket, which promoted Van der Waals interactions with Val104 and Phe294 (Figure 5A, mid and lower row). As for capnosane diterpene **2**, both C-4 and C-8 hydroxyls participated in hydrogen bonds with His164 and Glu166 (Figure 5B, upper row), respectively, while for compound **3**, its C-4 and C-8 hydroxyls participated in hydrogen bonds with LEU-287 and TYR-239 (Figure 5C, upper row), respectively. In addition, both capnosane diterpenes **2** and **3** laid in the hydrophobic pocket through Van der Waals interactions with a number of key amino acids (Figure 5B,C, mid and lower rows). The low binding affinities of compounds **1**–**3** (Table 2) revealed these three diterpenes were potential SARS-CoV-2 M^pro^ inhibitors.

## 3. Materials and Methods

### 3.1. General Experimental Procedures

Optical rotations were recorded on a Perkin-Elmer 241MC polarimeter. IR spectra were obtained on a Nicolet 6700 spectrometer (Thermo Scientific, Waltham, MA, USA). CD spectra were measured on a JASCO J-810 instrument. NMR spectra were measured on a Bruker DRX-500 or Bruker DRX-600 spectrometer (Bruker Biospin AG, Fällanden, Germany). Chemical shifts (*δ*) were reported in ppm with reference to the solvent signals, and coupling constants (*J*) were in Hz. ESIMS spectra were obtained on a Finngan-MAT-95 mass spectrometer. HRESIMS spectra were measured on an Agilent 1290-6545 UHPLC-QTOF mass spectrometer. Commercial silica gel (Qingdao Haiyang Chemical Group Co., Ltd., Qingdao, China, 200–300 and 400–600 mesh), Sephadex LH-20 gel (Amersham Biosciences, Piscataway, NJ, USA) were used for column chromatography, and precoated silica gel plates (Yan Tai Zi Fu Chemical Group Co., Yantai, China, G60 F-254) were used for analytical TLC. Reversed-phase (RP) HPLC was performed on an Agilent 1260 series liquid chromatography equipped with a DAD G1315D detector at 210 and 254 nm. A semi-preparative ODS-HG-5 column [5 µm, 250 × 9.4 mm] was employed for the purifications. All solvents used for column chromatography and HPLC were of analytical grade (Shanghai Chemical Reagents Co., Ltd., Shanghai, China) and chromatographic grade (Dikma Technologies Inc., Foothill Ranch, CA, USA), respectively.

### 3.2. Biological Material

The soft corals of *Sarcophyton mililatensis* were collected at a depth of −20 m by SCUBA diving from the coast of Xigu Island, Hainan Province, China, in May 2014. They were frozen immediately after collection, and identified by Prof. X.-B. Li from Hainan University. A voucher specimen (No. 14S-80) is available for inspection at Shanghai Institute of Materia Medica, Chinese Academy of Sciences.

### 3.3. Extraction and Isolation

The frozen animals (400 g, dry weight) were cut into pieces and extracted exhaustively with acetone at room temperature (3 × 1.5 L). The organic extract was evaporated to give a dark brown residue that was partitioned between Et_2_O and H_2_O. The upper layer was concentrated under reduced pressure to give a Et_2_O portion (13.5 g). The Et_2_O extract was separated into twenty-one fractions (A–U) by gradient silica gel column chromatography [0→100% Et_2_O (EE) in petroleum ether (PE)]. Fraction J was further purified by Sephadex LH-20 [PE/CH_2_Cl_2_/MeOH (2:1:1)], followed by silica gel column chromatography [PE/EE (2:1)] to give three subfractions. Subfraction J2D was further purified by RP-HPLC [MeCN/H_2_O (90:10), 3.0 mL/min] to give compound **2** (2.5 mg, *t*_R_ = 6.2 min). Similarly, subfraction J2F was subjected to RP-HPLC [MeCN/H_2_O (82:18), 3.0 mL/min] to yield compound **3** (3.1 mg, *t*_R_ = 8.6 min). Fraction P was further purified by Sephadex LH-20 [PE/CH_2_Cl_2_/MeOH (2:1:1)], followed by silica gel column chromatography [PE/acetone (3:1)] to afford three subfractions. Purification of subfraction P2C by RP-HPLC [MeOH/H_2_O (90:10), 3.0 mL/min] to give compound **2** (3.2 mg, *t*_R_ = 12.4 min).

### 3.4. Spectroscopic Data of Compounds

Mililatensol A (**1**): white powder; [α]D19 −51.8 (*c* 0.25, CHCl_3_); IR (KBr): *ν*_max_ 3282, 2952, 2932, 2852, 1436, 1385, 1196, 1180, 1131, 1076, 1042, 990, 635 cm^−1^; For ^1^H NMR (CDCl_3_, 600 MHz) and ^13^C NMR (CDCl_3_, 125 MHz) spectral data, see Table 1; HREIMS *m/z* 286.2293 (M^+^; calcd. for C_20_H_30_O, 286.2291).

Mililatensol B (**2**): colorless oil; [α]D19 −50.8 (*c* 0.2, CHCl_3_); IR (KBr): *ν*_max_ 3445, 2917, 2849, 1383, 1196, 1180, 1132, 1076 cm^−1^; For ^1^H NMR (CDCl_3_, 600 MHz) and ^13^C NMR (CDCl_3_, 125 MHz) spectral data, see Table 1; HRESIMS *m/z* 327.2297 ([M + Na]^+^; calcd. for C_20_H_32_NaO_2_, 327.2295).

Mililatensol C (**3**): colorless oil; [α]D19 −32.0 (*c* 0.25, CHCl_3_); IR (KBr): *ν*_max_ 3447, 2921, 2851, 1494, 1383, 1196, 1180, 1132, 1076 cm^−1^; For ^1^H NMR (CDCl_3_, 600 MHz) and ^13^C NMR (CDCl_3_, 125 MHz) spectral data, see Table 1; HRESIMS *m/z* 327.2298 ([M + Na]^+^; calcd. for C_20_H_32_NaO_2_, 327.2295).

### 3.5. Anti-Inflammatory Activity Assay

The murine macrophage cell line RAW264.7 was obtained from American Type Culture Collection (ATCC, Manassas, VA, USA). In the bioassay for anti-inflammation, cells were cultured in DMEM containing 10% FBS, 2 mmol/L L-glutamine, 100 μg/mL streptomycin, and 100 U/mL penicillin in a humidified incubator of 5% CO_2_ at 37 °C. For the cytotoxicity part, RAW264.7 cells were incubated with compounds or the media (0.125% DMSO in DMEM containing 10% FBS) for 24 h, respectively. CCK-8 reagents (20 μL per well) were added, and the OD values were collected after 1 h incubation at 450 nm (650 nm calibration) by a microplate reader (Molecular Devices, Sunnyvale, CA, USA). For the anti-inflammatory activity assay, RAW264.7 cells were incubated with compounds or the media (0.125% DMSO in DMEM containing 10% FBS), and then cells were primed with LPS (1 μg/mL) for 24 h. The supernatants were centrifuged and then measured using the mouse TNF-α ELISA kit. The CC_50_ and IC_50_ were estimated using the log (inhibitor) vs. normalized response non-linear fit (Graph Pad Prism 6.0, GraphPad Software, San Diego, CA, USA). Dexamethasone was used as a positive control.

### 3.6. Molecular Docking

AutoDock 4.2 and AutoDock Tools 1.5.7 software were downloaded from the official website (https://autodock.scripps.edu/ (accessed on 20 August 2022)), compounds **1**–**3** by Chem3D was optimized to export the mol2 format files, and the common crystal structure was obtained from the RCSB protein database (PDB ID: 6LU7). The 6LU7 receptor was imported into the software Pymol, and the 02J, 010, AVL, PJE groups contained in the 6LU7 receptor file could be deleted, where the water molecules could also be deleted in AutoDock, and the pdb file was finally exported. The resulting files were imported into AutoDock, hydrated, merged with non-polar hydrogen atoms, and saved as 6LU7.pdbqt. AutoDock 4.2 can choose flexible or rigid docking; this experiment adopted flexible docking. The AutoDock reads into the ligand and also hydrogenates the ligand to set it to a ligand. The Ligand subroutine in the AutoDock Tools 1.5.7 software can automatically detect the number of rotatable bonds that can rotate to dock with the receptor molecule during docking. The active site of 6LU7 was not detected, so the coordinate value was set to (−26.427, 12.578, 58.908) directly, and the lattice spacing of the docking parameter was set to 0.603Å.

## 4. Conclusions

In summary, the chemical study on the soft coral *S. mililatensis* led to the isolation and characterization of three uncommon diterpenes, mililatensols A–C (**1**–**3**), bearing rarely encountered sarsolenane and capnosane skeletons. As far as we know, this is the first record of sarsolenane and capnosane diterpenes from soft coral *S. mililatensis*. Moreover, it is interesting to note that this is the third report of the co-isolation of both types of diterpenes from one soft coral to date. These new findings indicated the rarely investigated soft coral *S. mililatensis* was a reservoir of structurally diverse terpenes. In the in vitro bioassay, none of the isolates exhibited obvious anti-inflammatory activity. The preliminary virtual screening by molecular docking experiments showed these three diterpenes exhibited potential inhibitory activities against SARS-CoV-2 M^pro^ inhibitors. Further studies on terpene biosynthetic gene clusters, biomimetic synthesis, and other biological assays will be carried out to realize the real ecological and/or biological roles played by these three interesting diterpenes during the life cycle of the soft corals and their potential medicinal application.

## Figures and Tables

**Table 1:** ^1^H NMR and ^13^C NMR data for compounds **1**–**3** ^a^.

No.	1		2		3	
	*δ*_H_ Mult. (*J*, Hz)	*δ*_C_, Type	*δ*_H_ Mult. (*J*, Hz)	*δ*_C_, Type	*δ*_H_ Mult. (*J*, Hz)	*δ*_C_, Type
1		132.5, C		142.8, C		143.0, C
2	3.73 td (3.4, 10.2)	37.5, CH	5.54 d (9.6)	130.0, CH	5.62 d (9.4)	129.2, CH
3	5.03 d (10.2)	128.1, CH	2.73 dd (9.6, 11.0)	51.2, CH	2.37 m	50.8, CH
4		130.0, C		82.2, C		83.0, C
5	2.03 m	38.2, CH_2_	1.76 m	39.6, CH_2_	1.70 m; 1.80 m	39.5, CH_2_
6	2.13 m	25.4, CH_2_	1.37 m; 1.71 m	23.6, CH_2_	1.30 m; 1.92 m	24.9, CH_2_
7	4.95 t (7.4)	127.6, CH	2.05 m	57.2, CH	2.50 m	57.9, CH
8		131.3, C		74.8, C		74.5, C
9	2.12 m; 2.41 d (12.4)	48.2, CH_2_	1.61 m; 1.86 m	34.2, CH_2_	1.59 m; 1.74 m	34.2, CH_2_
10	4.37 dt (2.8, 9.4)	68.4, CH	2.05 m; 2.34 m	23.0, CH_2_	2.01 m; 2.36 m	23.0, CH_2_
11	5.20 d (9.4)	126.8, CH	5.29 dd (5.4, 10.0)	127.9, CH	5.24 dd (5.3, 10.1)	128.2, CH
12		139.4, C		135.4, C		135.0, C
13	2.08 m; 2.28 m	36.8, CH_2_	1.94 m; 2.25 m	35.9, CH_2_	1.94 m; 2.24 m	35.6, CH_2_
14	2.10 m; 2.63 m	26.7, CH_2_	2.44 m; 2.81 dt (3.6,13.4)	27.4, CH_2_	2.48 m; 2.52 m	28.0, CH_2_
15		122.6, C		141.3, C		142.1, C
16	1.67 s	20.6, CH_3_	4.99 s; 5.09 s	112.8, CH_2_	5. 00 s; 5.08 s	113.0, CH_2_
17	1.69 s	20.2, CH_3_	1.91 s	21.9, CH_3_	1.91 s	22.0, CH_3_
18	1.64 s	16.3, CH_3_	1.12 s	24.2, CH_3_	1.22 s	26.8, CH_3_
19	1.64 s	18.0, CH_3_	1.16 s	31.9, CH_3_	1.19 s	32.3, CH_3_
20	2.02 m; 2.30 m	36.2, CH_2_	1.63 s	18.8, CH_3_	1.66 s	18.8, CH_3_

^a^ Recorded at 600 and 125 MHz for ^1^H and ^13^C in CDCl_3_, respectively. Assignments were deduced by analysis of 1D and 2D NMR spectra.

**Table 2:** In silico molecular docking binding affinities of compounds **1**–**3** to SARS-CoV-2 M^pro^ crystal structure (PDB: 6LU7).

Crystal Structure	Compound ID	Affinity Energy (kcal mol^−1^)
6LU7	**1**	−7.65
	**2**	−7.08
	**3**	−7.03

## Data Availability

Data are contained within the article or Supplementary Materials.

## Supplementary Materials

The following supporting information can be downloaded at: https://www.mdpi.com/article/10.3390/md20090566/s1. Figure S1: LREIMS and HREIMS spectra of compound **1**; Figure S2: ^1^H NMR spectrum (600 MHz) of compound **1** in CDCl_3_; Figure S3: ^13^C NMR (BB+DEPT) spectrum (125 MHz) of compound **1** in CDCl_3_; Figure S4: HSQC spectrum (600 MHz) of compound **1** in CDCl_3_; Figure S5: ^1^H–^1^H COSY spectrum (600 MHz) of compound **1** in CDCl_3_; Figure S6: HMBC spectrum (600 MHz) of compound **1** in CDCl_3_; Figure S7: NOESY spectrum (600 MHz) of compound **1** in CDCl_3_; Figure S8: IR spectrum of compound **1**; Figure S9: HRESIMS spectrum of compound **2**; Figure S10: ^1^H NMR spectrum (600 MHz) of compound **2** in CDCl_3_; Figure S11: ^13^C NMR (BB+DEPT) spectrum (125 MHz) of compound **2** in CDCl_3_; Figure S12: HSQC spectrum (600 MHz) of compound **2** in CDCl_3_; Figure S13: ^1^H–^1^H COSY spectrum (600 MHz) of compound **2** in CDCl_3_; Figure S14: HMBC spectrum (600 MHz) of compound **2** in CDCl_3_; Figure S15: NOESY spectrum (600 MHz) of compound **2** in CDCl_3_; Figure S16: IR spectrum of compound **2**; Figure S17: HRESIMS spectrum of compound **3**; Figure S18: ^1^H NMR spectrum (600 MHz) of compound **3** in CDCl_3_; Figure S19: ^13^C NMR (BB+DEPT) spectrum (125 MHz) of compound **3** in CDCl_3_; Figure S20: HSQC spectrum (600 MHz) of compound **3** in CDCl_3_; Figure S21: ^1^H–^1^H COSY spectrum (600 MHz) of compound **3** in CDCl_3_; Figure S22: HMBC spectrum (600 MHz) of compound **3** in CDCl_3_; Figure S23: NOESY spectrum (600 MHz) of compound **3** in CDCl_3_; Figure S24: IR spectrum of compound **3**; Figure S25: Re-optimized conformers above 1% population (OPLS_2005) of (3*S,*4*S,*7*R*,8*S*)-**2** calculated at the B3LYP/6-311G(d,p) level with IEFPCM solvent model for methanol; Table S1: Cartesian coordinates for the re-optimized conformers of compound **2** at the B3LYP/6-311G(d,p) level with IEFPCM solvent model for methanol.
