# Supplementary material for: Mililatensols A–C, New Records of Sarsolenane and Capnosane Diterpenes from Soft Coral Sarcophyton mililatensis
Source: Mar Drugs. 2022 Sep 6;20(9):566. doi: 10.3390/md20090566 (PMC9503382; doi:10.3390/md20090566)
Supplement: Supplementary file 1 [file marinedrugs-20-00566-s001.zip › marinedrugs-1889372-supplementary.pdf]

Supplementary Information

Mililatensols A–C, New Records of Sarsolenane and Capnosane Diterpenes from Soft Coral *Sarcophyton mililatensis*

Qing Bu [1,†], Min Yang [2,†], Xian-Yun Yan [1], Song-Wei Li [2], Zeng-Yue Ge [1], Ling Zhang [1], Li-Gong Yao [2], Yue-Wei Guo [2,3,4,5,]* and Lin-Fu Liang [1,]*

[1]College of Materials Science and Engineering, Central South University of Forestry and Technology, 498 South Shaoshan Road, Changsha 410004, China

[2]State Key Laboratory of Drug Research, Shanghai Institute of Materia Medica, Chinese Academy of Sciences, 555, Zu Chong Zhi Road, Zhangjiang Hi-Tech Park, Shanghai 201203, China

[3]Shandong Laboratory of Yantai Drug Discovery, Bohai rim Advanced Research Institute for Drug Discovery, Yantai 264117, China

[4]Open Studio for Druggability Research of Marine Natural Products, Pilot National Laboratory for Marine Science and Technology (Qingdao), 1 Wenhai Road, Aoshanwei, Jimo, Qingdao 266237, China

[5]Collaborative Innovation Center of Yangtze River Delta Region Green Pharmaceuticals and College of Pharmaceutical Science, Zhejiang University of Technology, Hangzhou 310014, China

*****Correspondence: ywguo@simm.ac.cn (Y.-W.G.); lianglinfu@csuft.edu.cn (L.-F.L.)

[†]These two authors contributed equally to this article.

Table of contents

**Figure S1.**	LREIMS and HREIMS spectra of compound **1**
**Figure S2.**	^{1}H NMR spectrum (600 MHz) of compound **1** in $CDCl_3$
**Figure S3.**	^{13}C NMR (BB+DEPT) spectrum (125 MHz) of compound **1** in $CDCl_3$
**Figure S4.**	HSQC spectrum (600 MHz) of compound **1** in $CDCl_3$
**Figure S5.**	^{1}H–^{1}H COSY spectrum (600 MHz) of compound **1** in $CDCl_3$
**Figure S6.**	HMBC spectrum (600 MHz) of compound **1** in $CDCl_3$
**Figure S7.**	NOESY spectrum (600 MHz) of compound **1** in $CDCl_3$
**Figure S8.**	IR spectrum of compound **1**
**Figure S9.**	HRESIMS spectrum of compound **2**
**Figure S10.**	^{1}H NMR spectrum (600 MHz) of compound **2** in $CDCl_3$
**Figure S11.**	^{13}C NMR (BB+DEPT) spectrum (125 MHz) of compound **2** in $CDCl_3$
**Figure S12.**	HSQC spectrum (600 MHz) of compound **2** in $CDCl_3$
**Figure S13.**	^{1}H–^{1}H COSY spectrum (600 MHz) of compound **2** in $CDCl_3$
**Figure S14.**	HMBC spectrum (600 MHz) of compound **2** in $CDCl_3$
**Figure S15.**	NOESY spectrum (600 MHz) of compound **2** in $CDCl_3$
**Figure S16.**	IR spectrum of compound **2**
**Figure S17.**	HRESIMS spectrum of compound **3**
**Figure S18.**	^{1}H NMR spectrum (600 MHz) of compound **3** in $CDCl_3$
**Figure S19.**	^{13}C NMR (BB+DEPT) spectrum (125 MHz) of compound **3** in $CDCl_3$
**Figure S20.**	HSQC spectrum (600 MHz) of compound **3** in $CDCl_3$
**Figure S21.**	^{1}H–^{1}H COSY spectrum (600 MHz) of compound **3** in $CDCl_3$
**Figure S22.**	HMBC spectrum (600 MHz) of compound **3** in $CDCl_3$
**Figure S23.**	NOESY spectrum (600 MHz) of compound **3** in $CDCl_3$
**Figure S24.**	IR spectrum of compound **3**
**Figure S25.**	Re-optimized conformers above 1% population (OPLS_2005) of (3*S*,4*S*,7*R*,8*S*)-**2** calculated at the B3LYP/6-311G(d,p) level with IEFPCM solvent model for methanol.
**Table S1.**	Cartesian coordinates for the re-optimized conformers of compound **2** at the B3LYP/6-311G(d,p) level with IEFPCM solvent model for methanol.

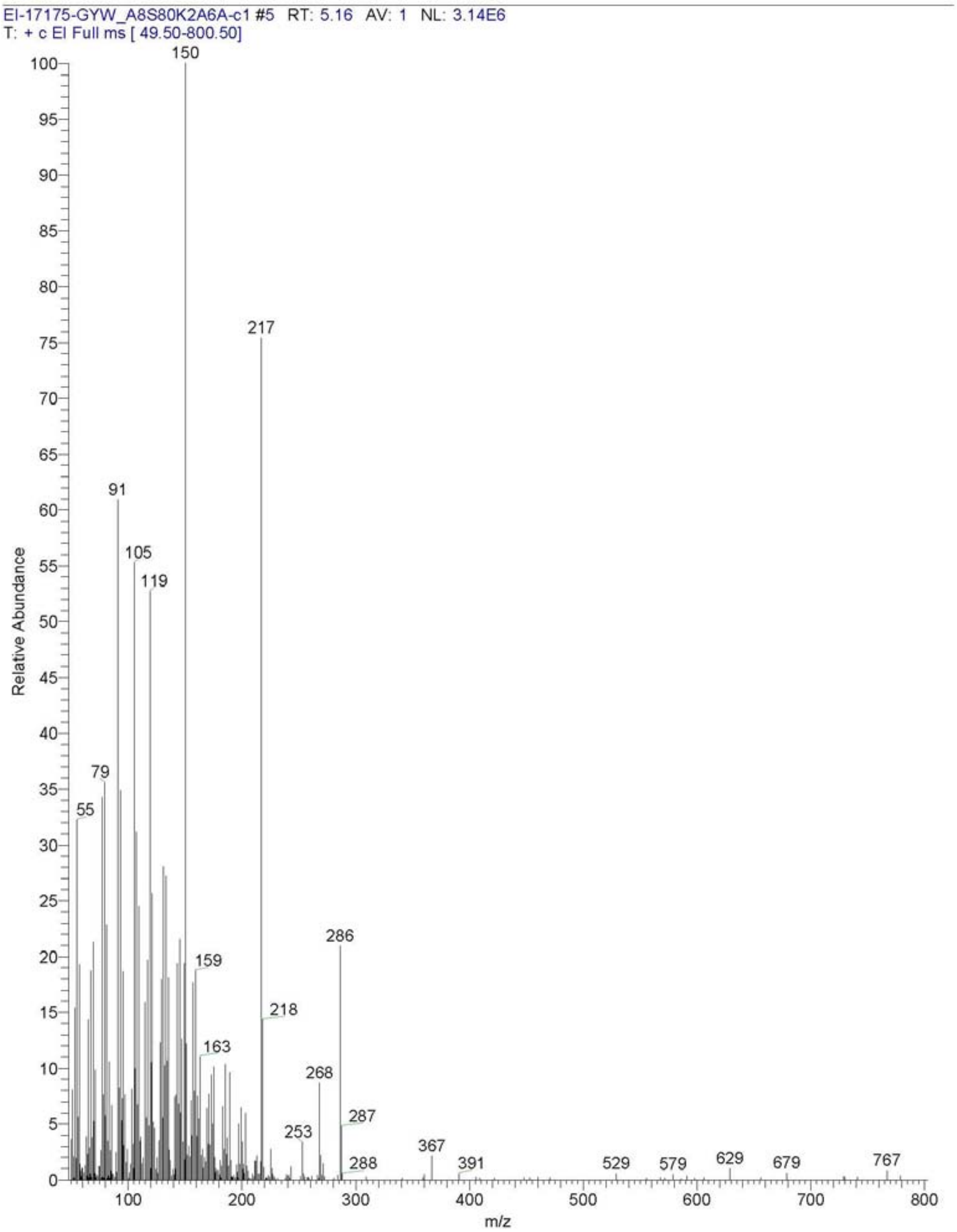
EI-17175-GYW_A8S80K2A6A-c1 #5 RT: 5.16 AV: 1 NL: 3.14E6
T: + c EI Full ms [49.50-800.50]
Relative Abundance
m/z
150
217
91
105
119
79
55
159
218
163
286
268
287
253
288
367
391
529
579
629
679
767

D:\data\...\EI-17175-GYW_A8S80K2A6A-c1 3/24/2017 3:01:40 PM

EI-17175-GYW_A8S80K2A6A-c1#7 RT: 5.58
T: + c EI Full ms [49.50-800.50]
m/z= 85.3570-302.7305

m/z	Intensity	Relative	Theo. Mass	Delta (mmu)	RDB equiv.	Composition
134.1088	205907.0	13.49	134.1090	-0.21	4.0	$C_{10}H_{14}$
135.0811	318112.0	20.84	135.0804	0.70	4.5	$C_{9}H_{11}O_{1}$
135.1173	224538.0	14.71	135.1168	0.52	3.5	$C_{10}H_{15}$
141.0702	166942.0	10.93	141.0699	0.35	7.5	$C_{11}H_{9}$
142.0772	155249.0	10.17	142.0777	-0.45	7.0	$C_{11}H_{10}$
143.0858	376350.0	24.65	143.0855	0.31	6.5	$C_{11}H_{11}$
144.0926	121649.0	7.97	144.0934	-0.74	6.0	$C_{11}H_{12}$
145.1016	422125.0	27.65	145.1012	0.42	5.5	$C_{11}H_{13}$
146.1074	111819.0	7.32	146.1090	-1.64	5.0	$C_{11}H_{14}$
147.0811	185894.0	12.18	147.0804	0.62	5.5	$C_{10}H_{11}O_{1}$
147.1175	194310.0	12.73	147.1168	0.69	4.5	$C_{11}H_{15}$
148.0873	87181.0	5.71	148.0883	-0.97	5.0	$C_{10}H_{12}O_{1}$
148.1241	45774.0	3.00	148.1247	-0.51	4.0	$C_{11}H_{16}$
149.0964	139284.0	9.12	149.0961	0.35	4.5	$C_{10}H_{13}O_{1}$
149.1331	57660.0	3.78	149.1325	0.63	3.5	$C_{11}H_{17}$
150.1044	1526699.0	100.00	150.1039	0.52	4.0	$C_{10}H_{14}O_{1}$
152.0621	56022.0	3.67	152.0621	0.06	9.0	$C_{12}H_{8}$
153.0688	68357.0	4.48	153.0699	-1.12	8.5	$C_{12}H_{9}$
154.0774	49436.0	3.24	154.0777	-0.32	8.0	$C_{12}H_{10}$
155.0857	123383.0	8.08	155.0855	0.21	7.5	$C_{12}H_{11}$
156.0931	76291.0	5.00	156.0934	-0.25	7.0	$C_{12}H_{12}$
157.1026	307061.0	20.11	157.1012	1.42	6.5	$C_{12}H_{13}$
158.1084	155667.0	10.20	158.1090	-0.65	6.0	$C_{12}H_{14}$
159.1156	298709.0	19.57	159.1168	-1.20	5.5	$C_{12}H_{15}$
160.1223	64341.0	4.21	160.1247	-2.38	5.0	$C_{12}H_{16}$
161.0958	122002.0	7.99	161.0961	-0.30	5.5	$C_{11}H_{13}O_{1}$
161.1317	71633.0	4.69	161.1325	-0.81	4.5	$C_{12}H_{17}$
162.1026	87984.0	5.76	162.1039	-1.29	5.0	$C_{11}H_{14}O_{1}$
163.1111	181461.0	11.89	163.1117	-0.65	4.5	$C_{11}H_{15}O_{1}$
165.0687	61129.0	4.00	165.0699	-1.21	9.5	$C_{13}H_{9}$
169.1011	127174.0	8.33	169.1012	-0.07	7.5	$C_{13}H_{13}$
170.1075	68710.0	4.50	170.1090	-1.55	7.0	$C_{13}H_{14}$
171.1168	161095.0	10.55	171.1168	0.02	6.5	$C_{13}H_{15}$
172.1222	42819.0	2.80	172.1247	-2.47	6.0	$C_{13}H_{16}$
173.1320	151137.0	9.90	173.1325	-0.49	5.5	$C_{13}H_{17}$
174.1389	79600.0	5.21	174.1403	-1.38	5.0	$C_{13}H_{18}$
175.1123	141918.0	9.30	175.1117	0.53	5.5	$C_{12}H_{15}O_{1}$
175.1472	55347.0	3.63	175.1481	-0.93	4.5	$C_{13}H_{19}$
183.1162	115899.0	7.59	183.1168	-0.59	7.5	$C_{14}H_{15}$
184.1238	45068.0	2.95	184.1247	-0.80	7.0	$C_{14}H_{16}$
185.1320	148471.0	9.72	185.1325	-0.47	6.5	$C_{14}H_{17}$
187.1479	57692.0	3.78	187.1481	-0.22	5.5	$C_{14}H_{19}$
189.1640	124764.0	8.17	189.1638	0.17	4.5	$C_{14}H_{21}$
197.1317	88144.0	5.77	197.1325	-0.77	7.5	$C_{15}H_{17}$
199.1484	94248.0	6.17	199.1481	0.30	6.5	$C_{15}H_{19}$
203.1438	70252.0	4.60	203.1430	0.72	5.5	$C_{14}H_{19}O_{1}$
217.1590	923722.0	60.50	217.1587	0.34	5.5	$C_{15}H_{21}O_{1}$
218.1638	154125.0	10.10	218.1665	-2.71	5.0	$C_{15}H_{22}O_{1}$
225.1638	53002.0	3.47	225.1638	-0.03	7.5	$C_{17}H_{21}$
253.1936	53002.0	3.47	253.1951	-1.44	7.5	$C_{19}H_{25}$
268.2199	109892.0	7.20	268.2186	1.37	7.0	$C_{20}H_{28}$
286.2293	219462.0	14.37	286.2291	0.23	6.0	$C_{20}H_{30}O_{1}$

Figure S1. LREIMS and HREIMS spectra of compound **1**

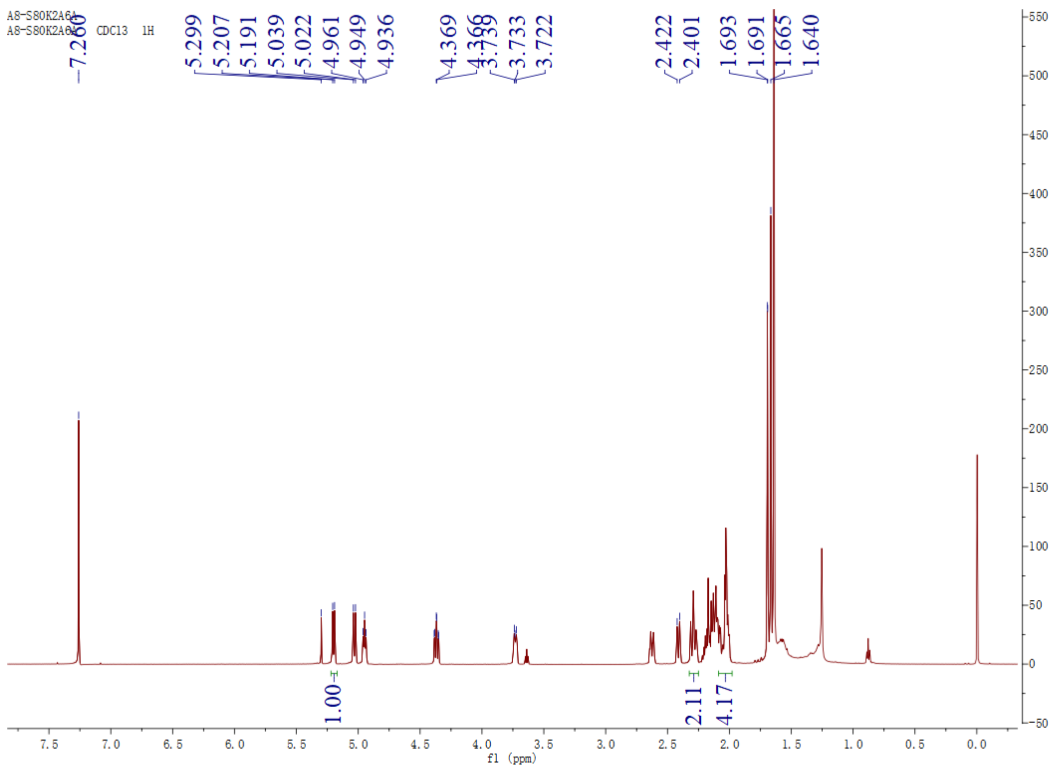

Figure S2. ^{1}H NMR spectrum (600 MHz) of compound **1** in $CDCl_3$

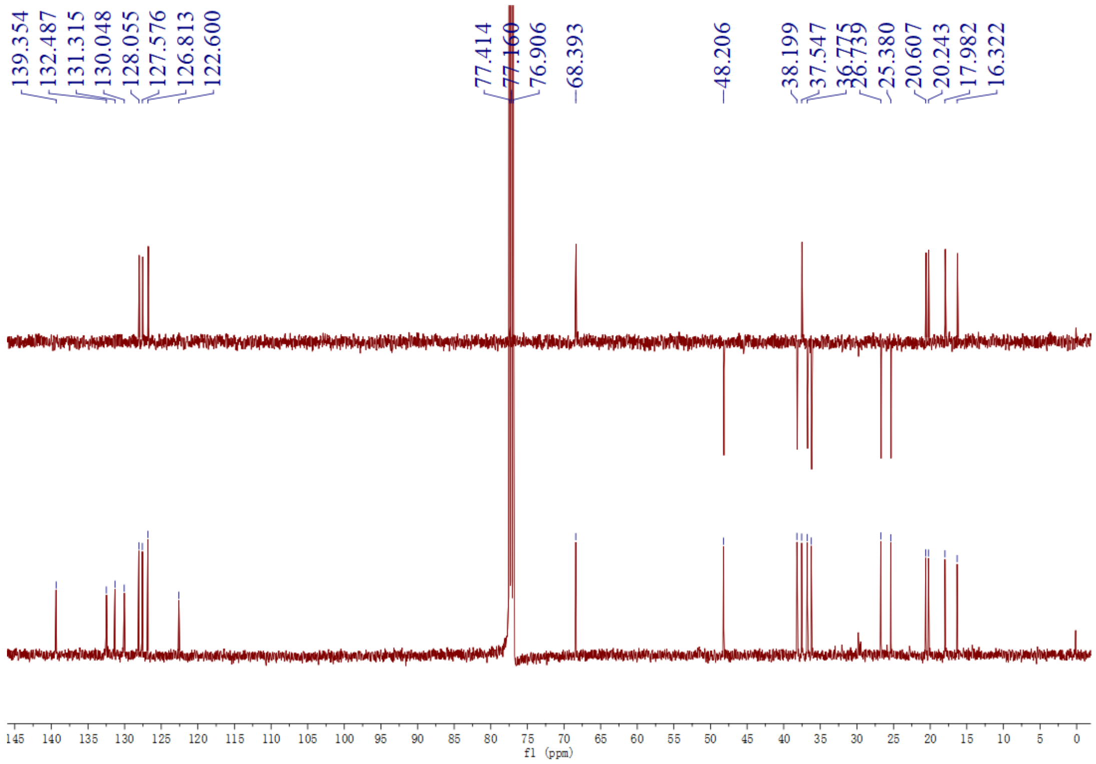

Figure S3. ^{13}C NMR (BB+DEPT) spectrum (125 MHz) of compound **1** in $CDCl_3$

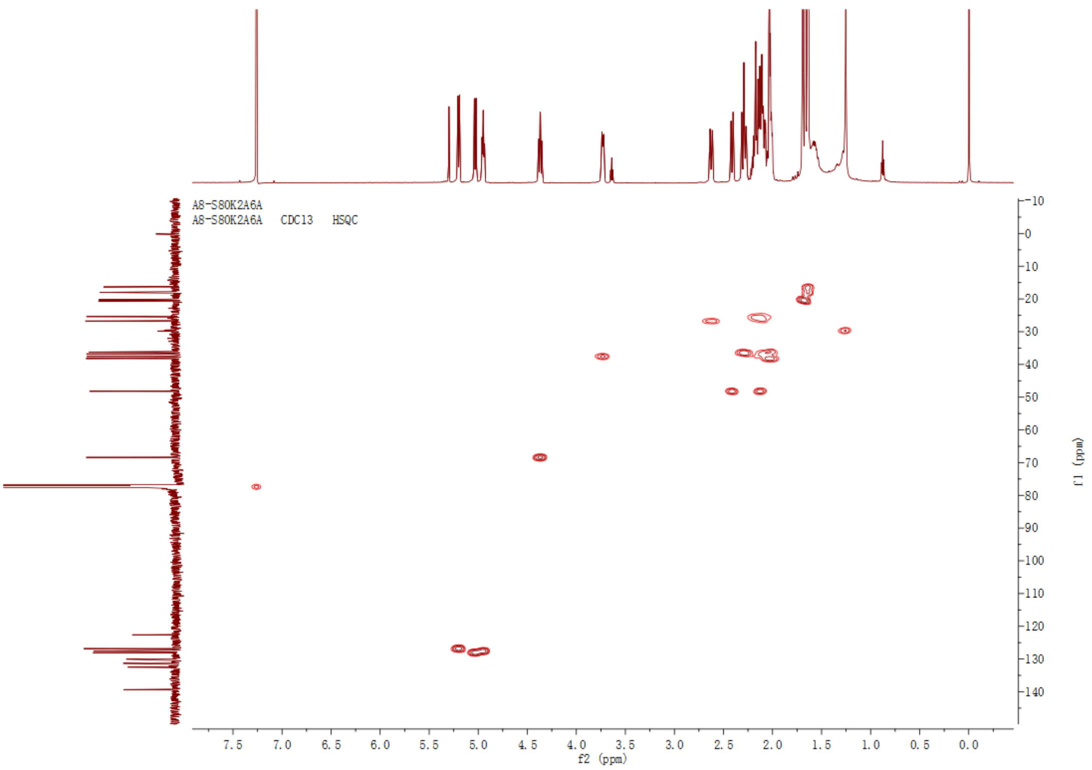

Figure S4. HSQC spectrum (600 MHz) of compound **1** in $CDCl_3$

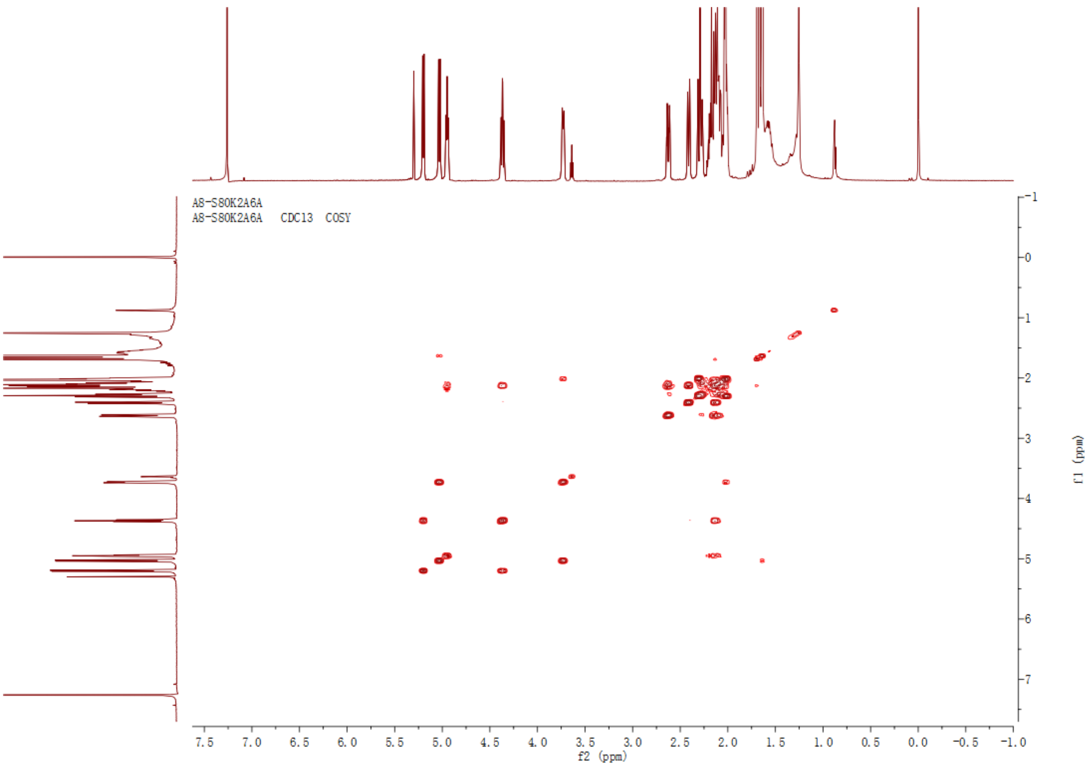

Figure S5. ^1H–^1H COSY spectrum (600 MHz) of compound **1** in $CDCl_3$

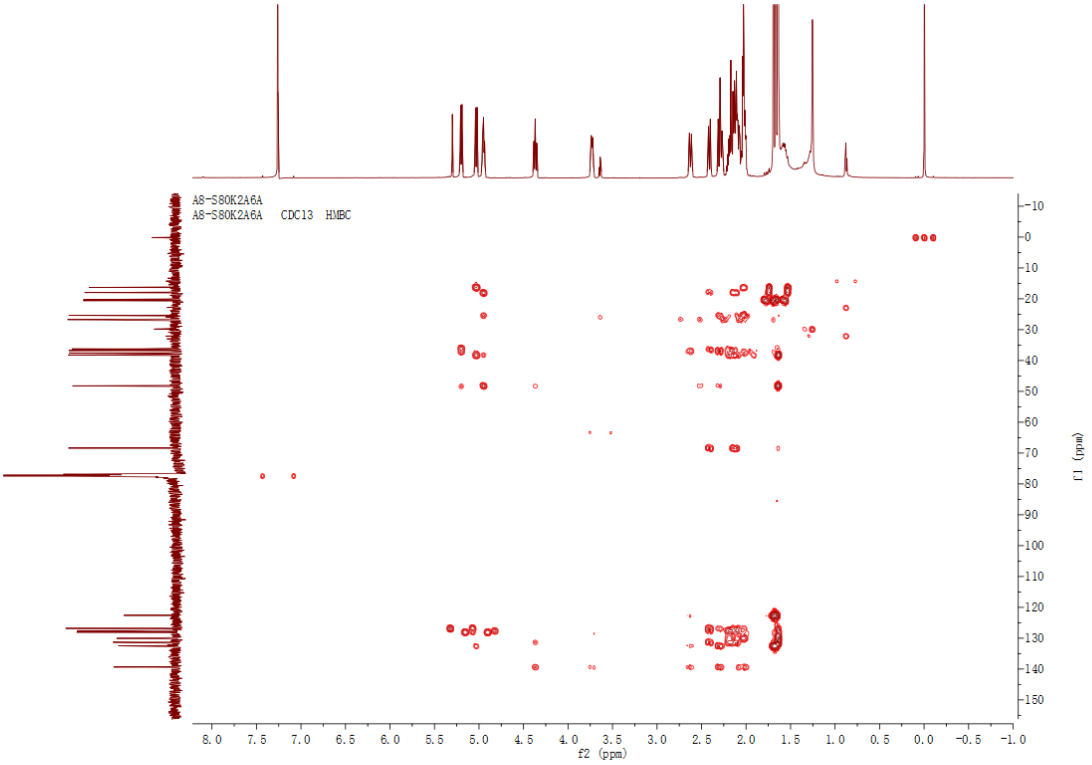

Figure S6. HMBC spectrum (600 MHz) of compound **1** in $CDCl_3$

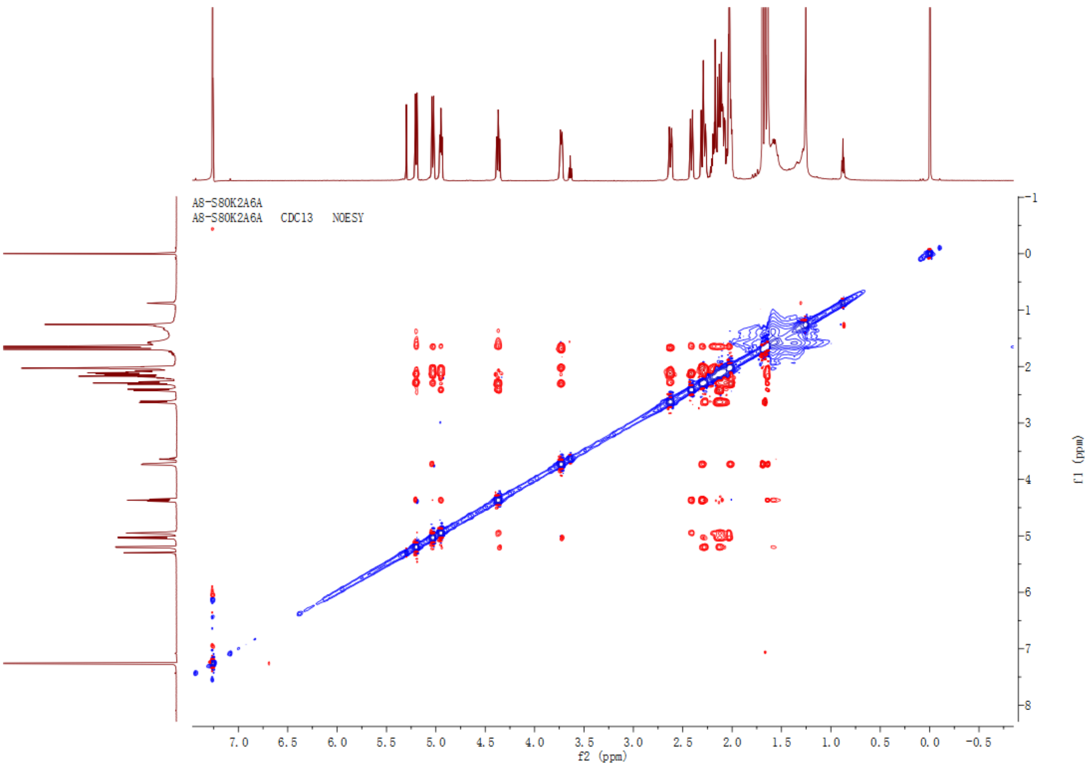

Figure S7. NOESY spectrum (600 MHz) of compound **1** in $CDCl_3$

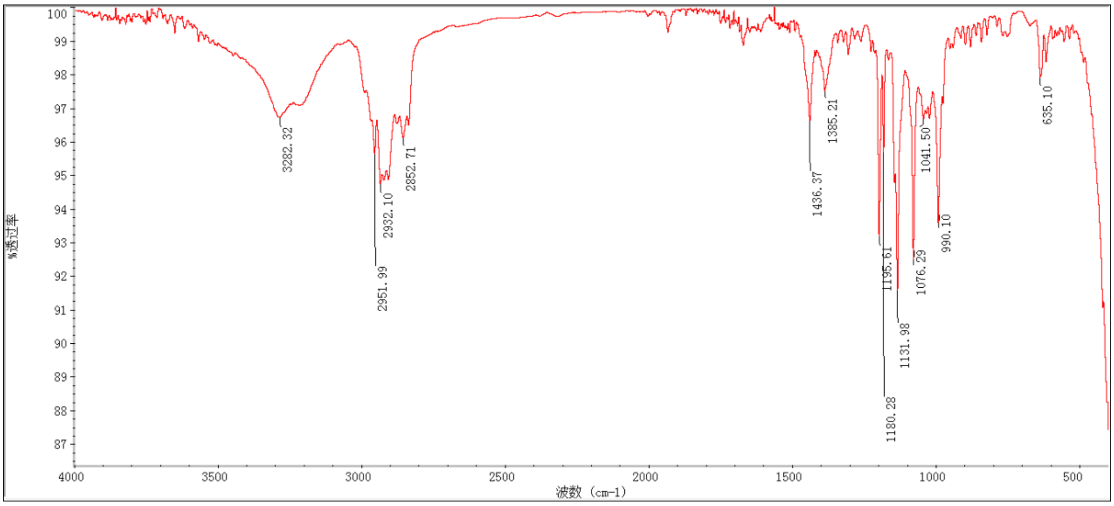

Figure S8. IR spectrum of compound **1**

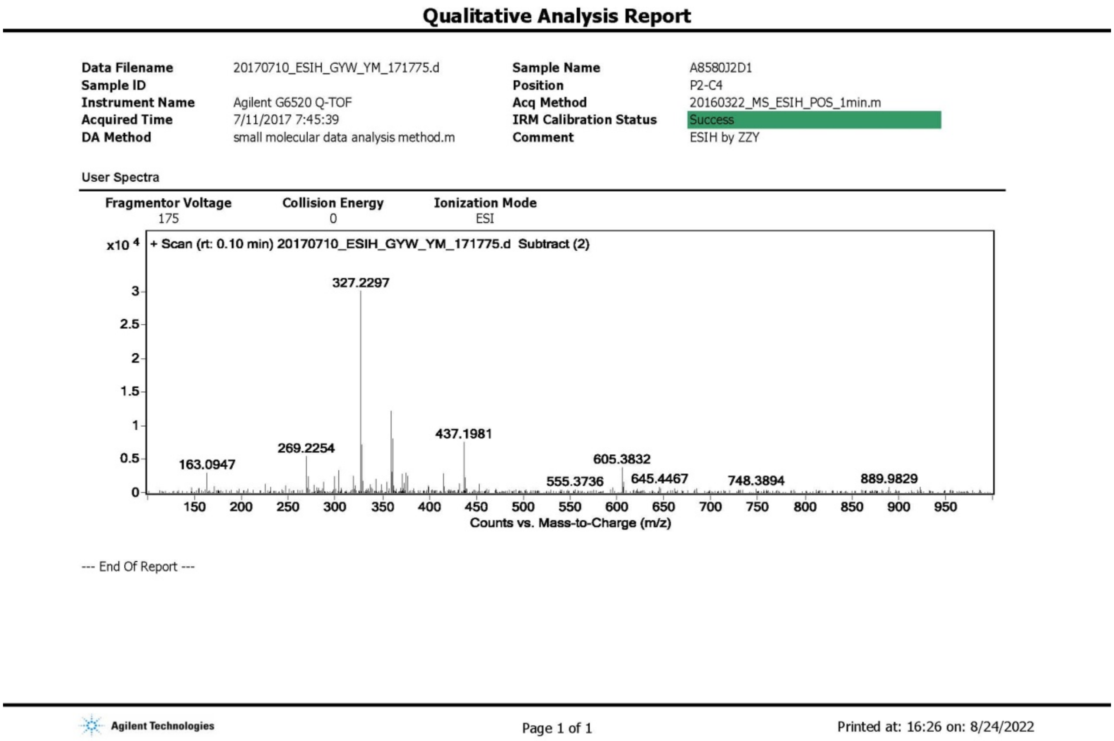

Figure S9. HRESIMS spectrum of compound **2**

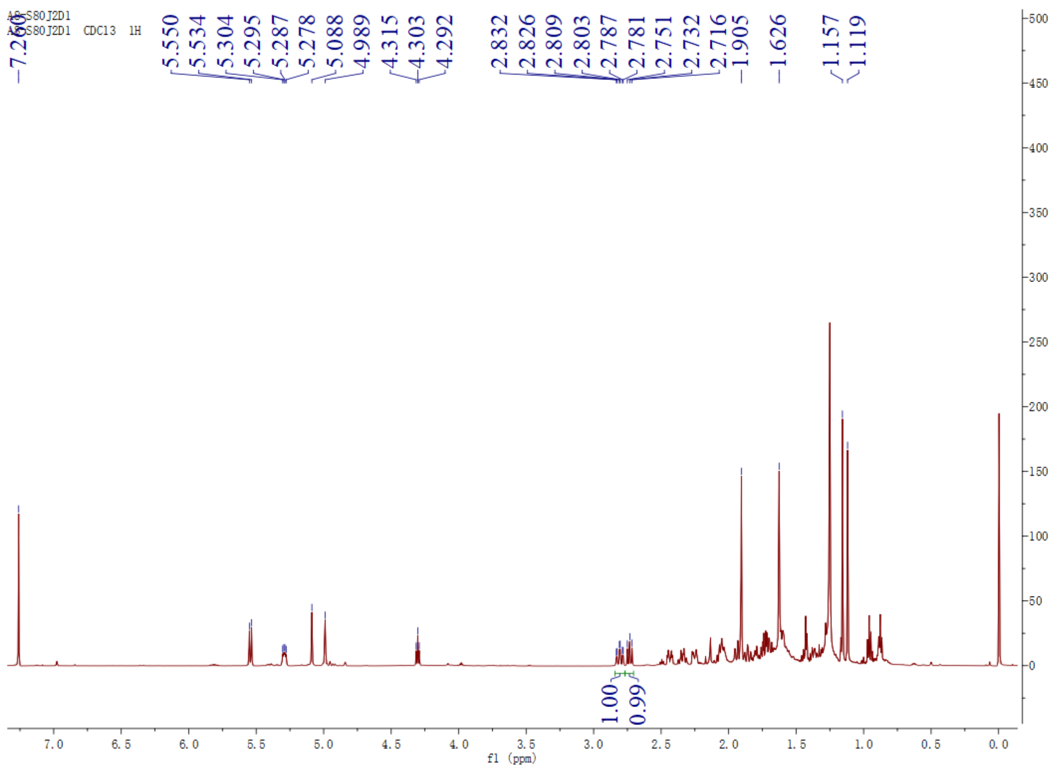

Figure S10. ^{1}H NMR spectrum (600 MHz) of compound **2** in $CDCl_3$

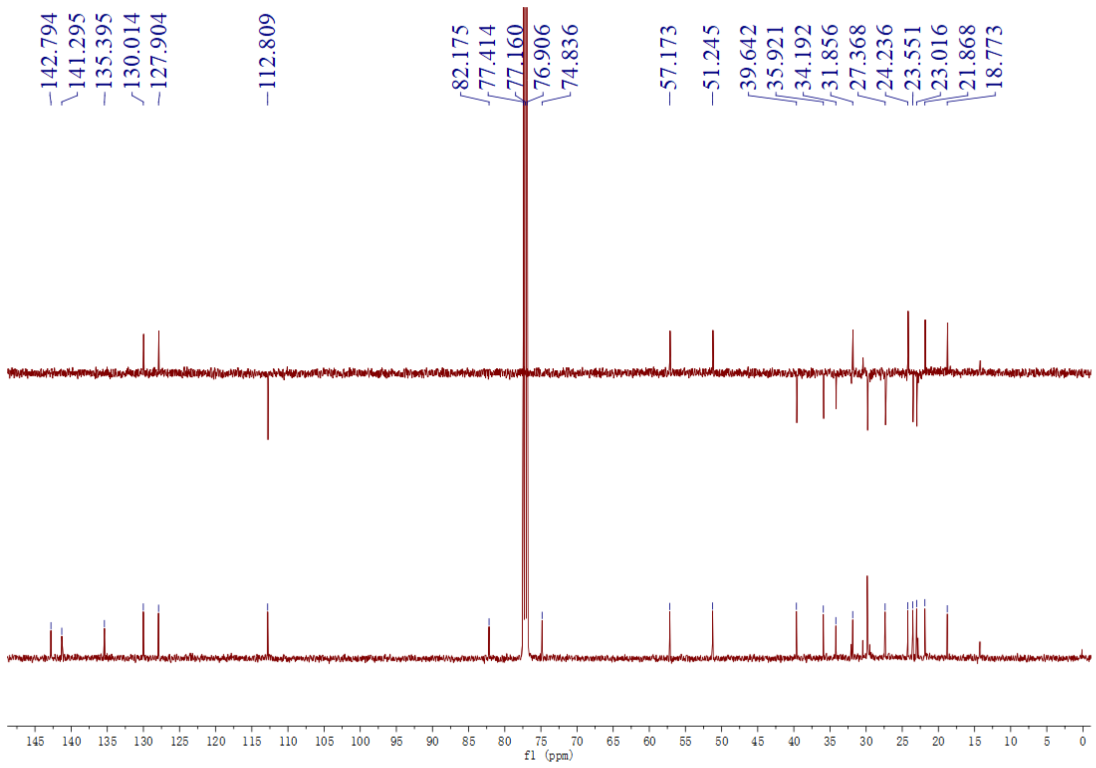

Figure S11. ^{13}C NMR (BB+DEPT) spectrum (125 MHz) of compound **2** in $CDCl_3$

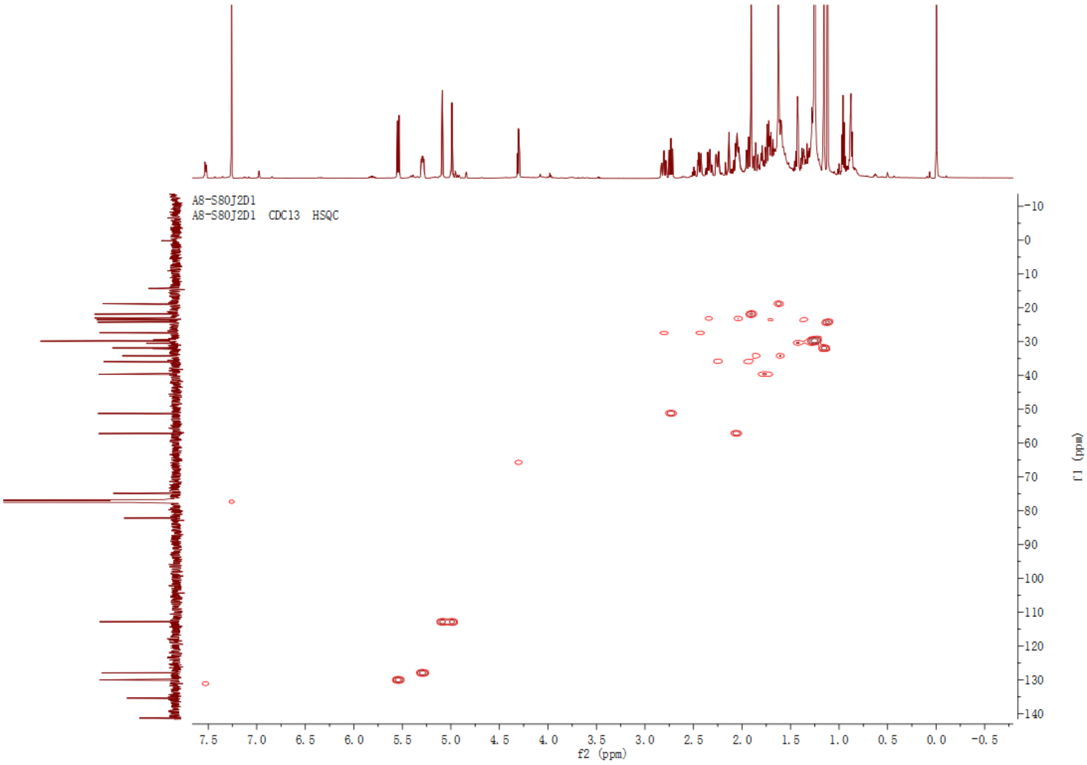

Figure S12. HSQC spectrum (600 MHz) of compound **2** in $CDCl_3$

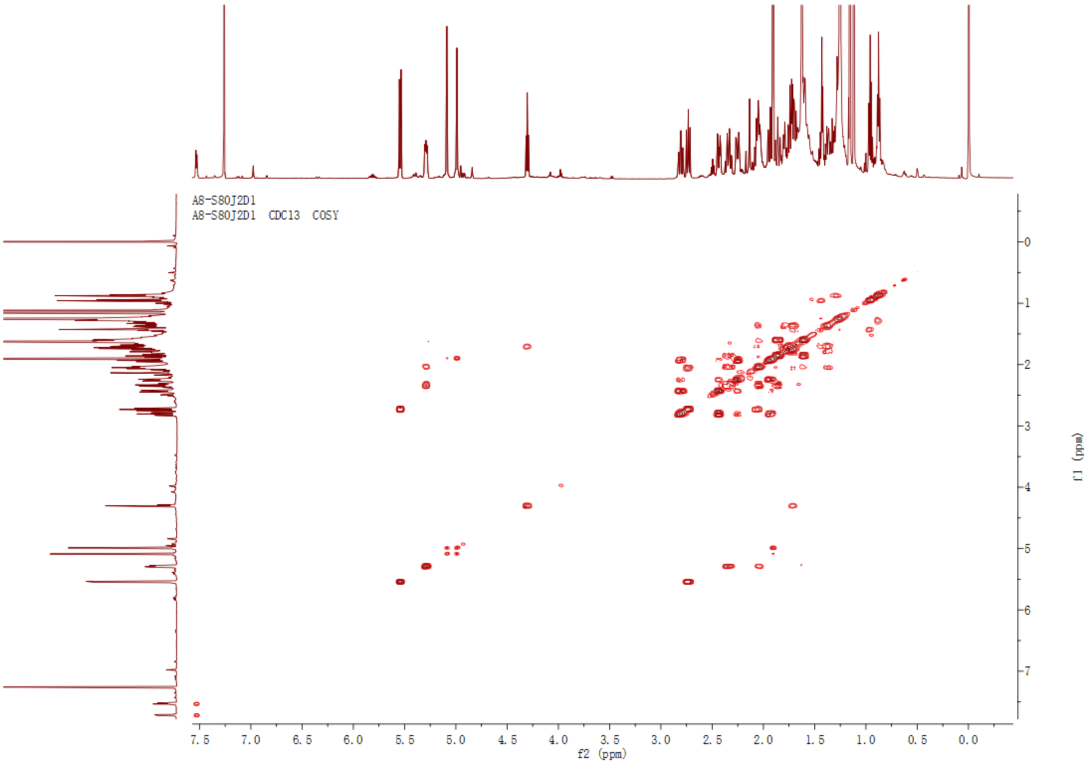

Figure S13. ^1H–^1H COSY spectrum (600 MHz) of compound **2** in $CDCl_3$

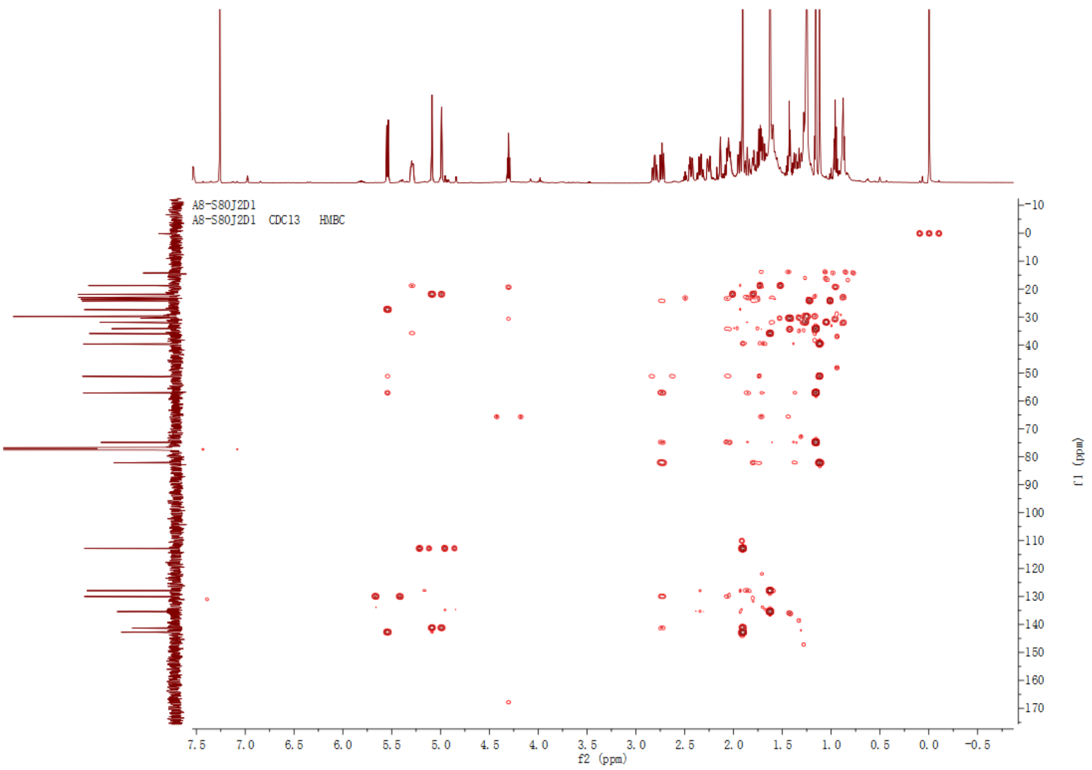

Figure S14. HMBC spectrum (600 MHz) of compound **2** in $CDCl_3$

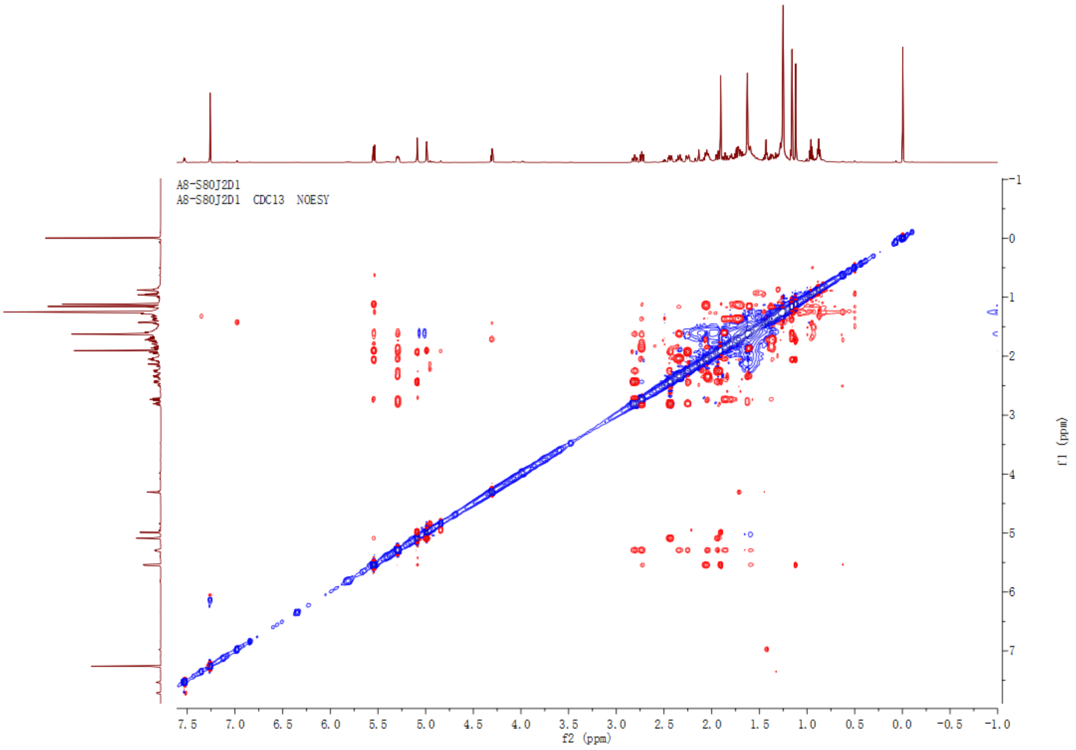

Figure S15. NOESY spectrum (600 MHz) of compound **2** in $CDCl_3$

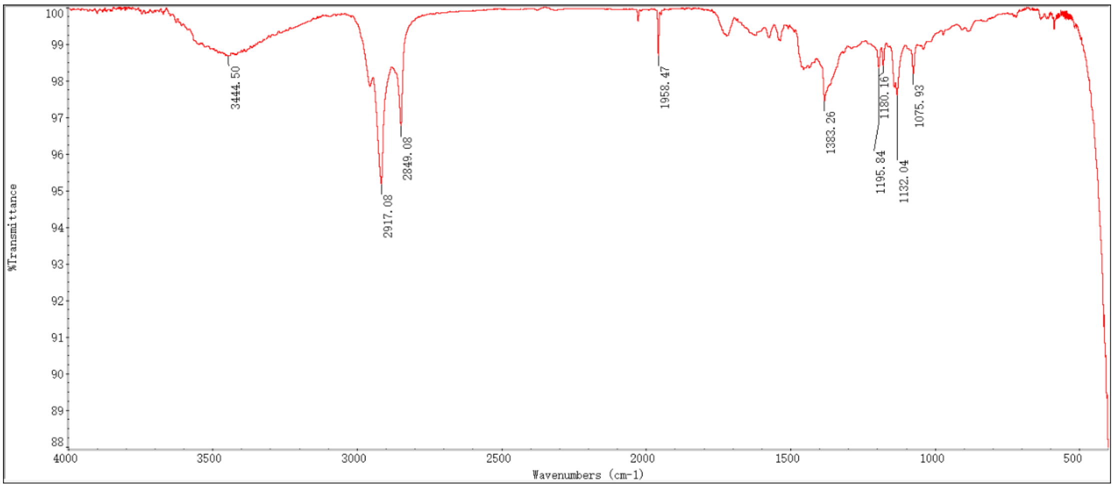

Figure S16. IR spectrum of compound **2**

Qualitative Analysis Report

**Data Filename**	20170710_ESIH_GYW_YM_171777.d	**Sample Name**	A8580J2F2
**Sample ID**		**Position**	P2-C6
**Instrument Name**	Agilent G6520 Q-TOF	**Acq Method**	20160322_MS_ESIH_POS_1min.m
**Acquired Time**	7/11/2017 7:49:20	**IRM Calibration Status**	Success
**DA Method**	small molecular data analysis method.m	**Comment**	ESIH by ZZY

User Spectra

Fragmentor Voltage	Collision Energy	Ionization Mode
175	0	ESI

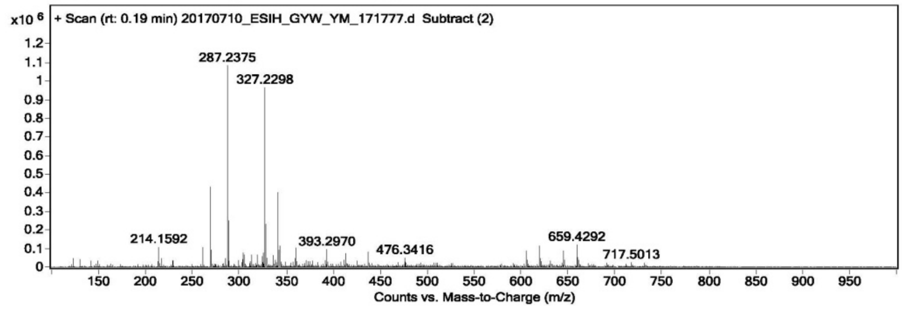

--- End Of Report ---

Agilent Technologies Page 1 of 1 Printed at: 16:31 on: 8/24/2022

Figure S17. HRESIMS spectrum of compound **3**

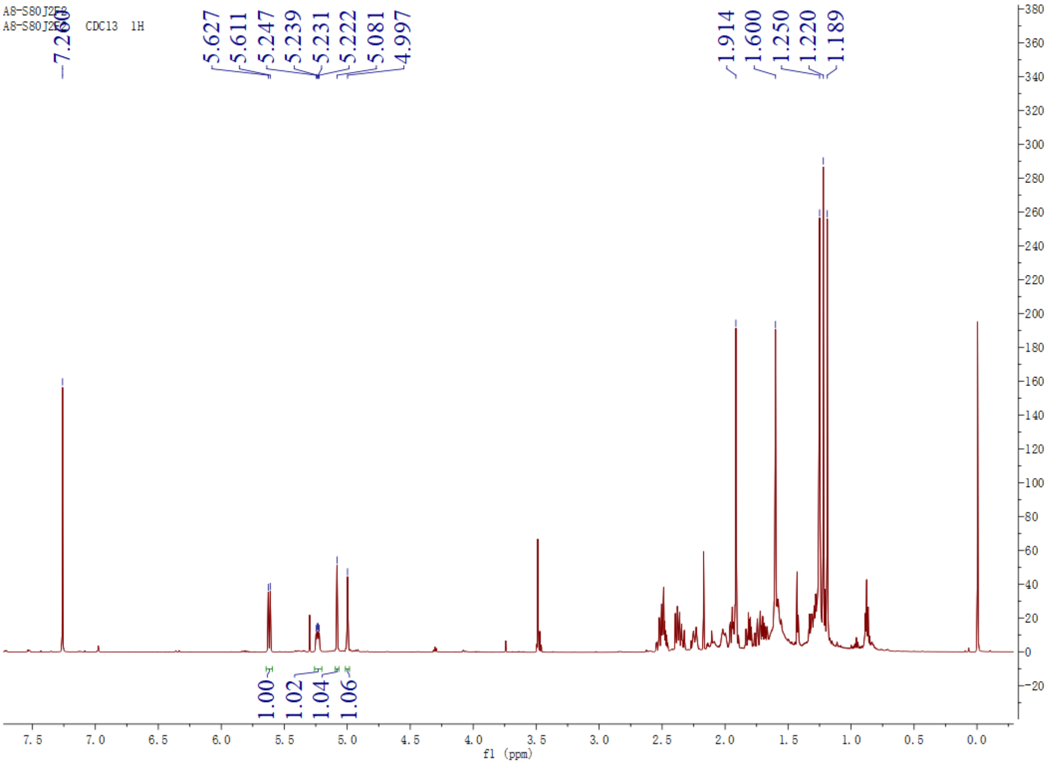

Figure S18. ^{1}H NMR spectrum (600 MHz) of compound **3** in $CDCl_3$

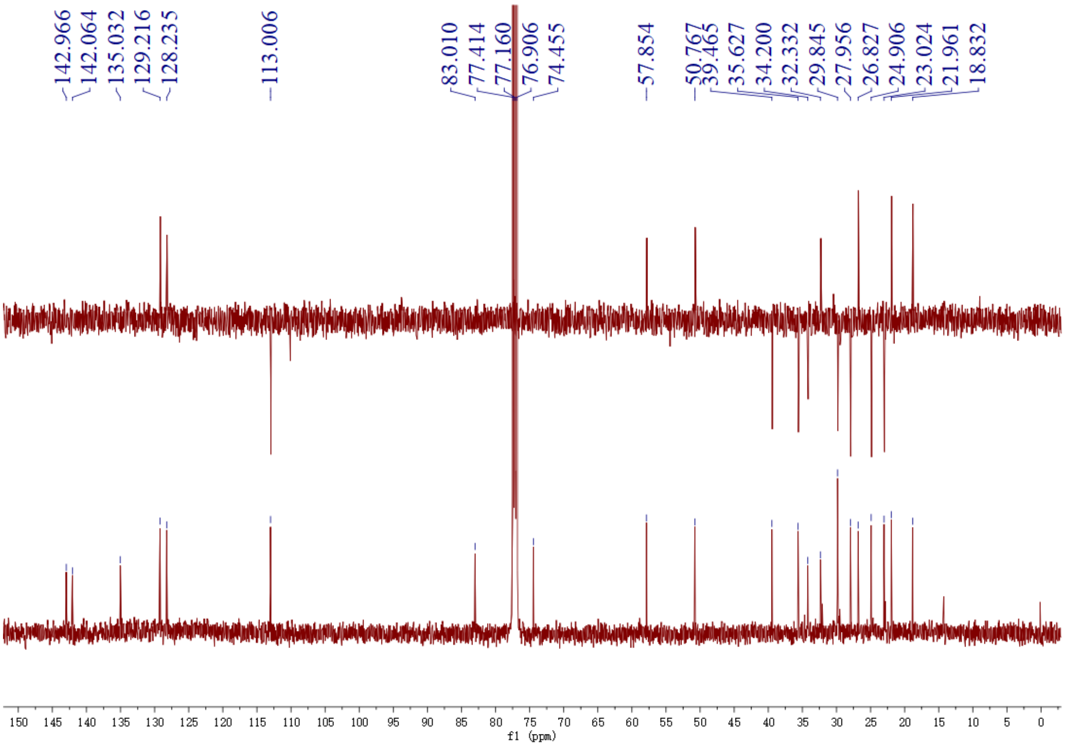

Figure S19. ^{13}C NMR (BB+DEPT) spectrum (125 MHz) of compound **3** in $CDCl_3$

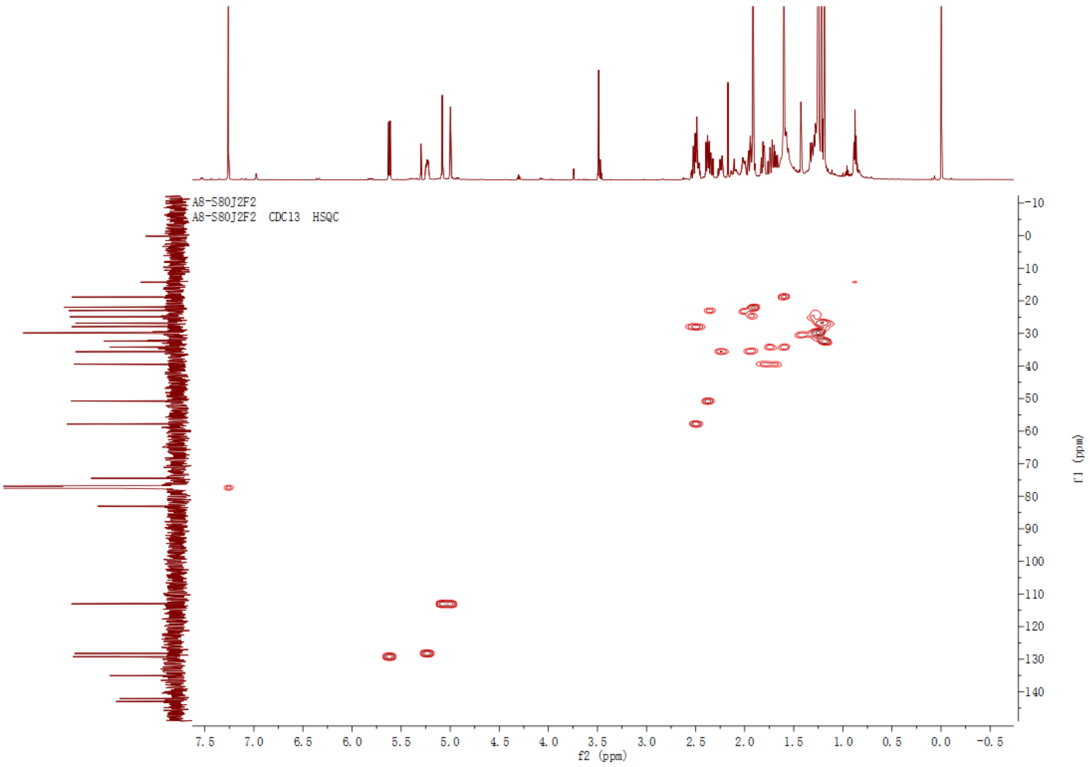

Figure S20. HSQC spectrum (600 MHz) of compound **3** in $CDCl_3$

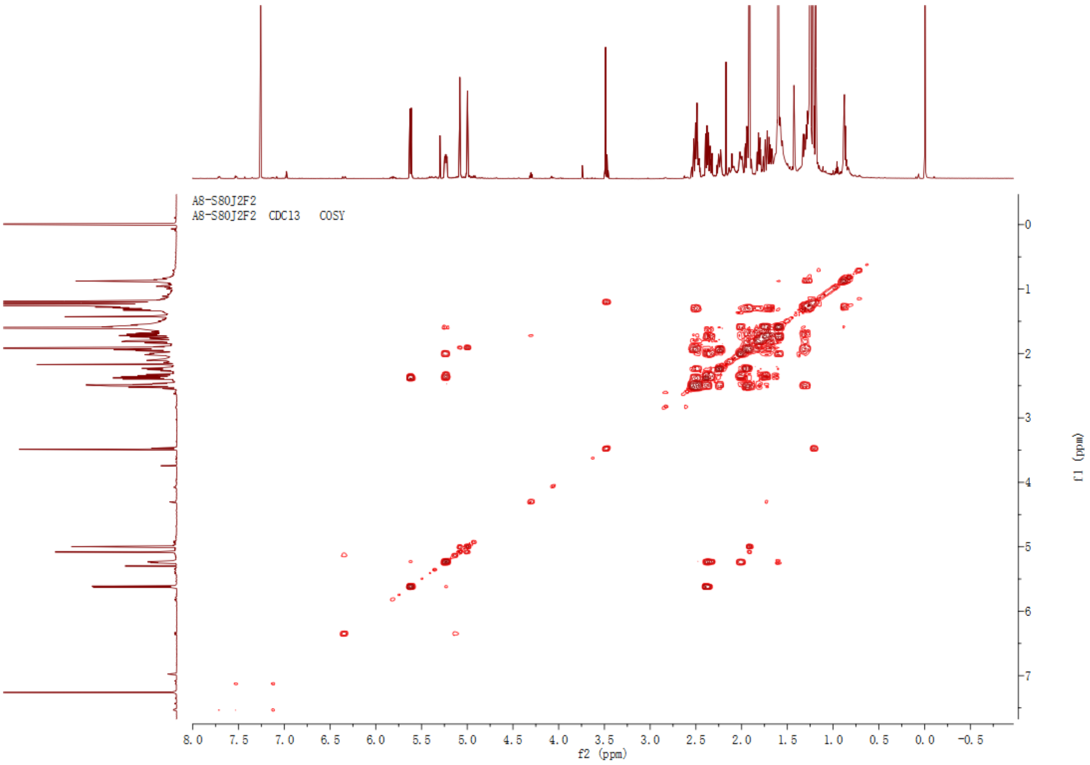

Figure S21. ^{1}H–^{1}H COSY spectrum (600 MHz) of compound **3** in $CDCl_3$

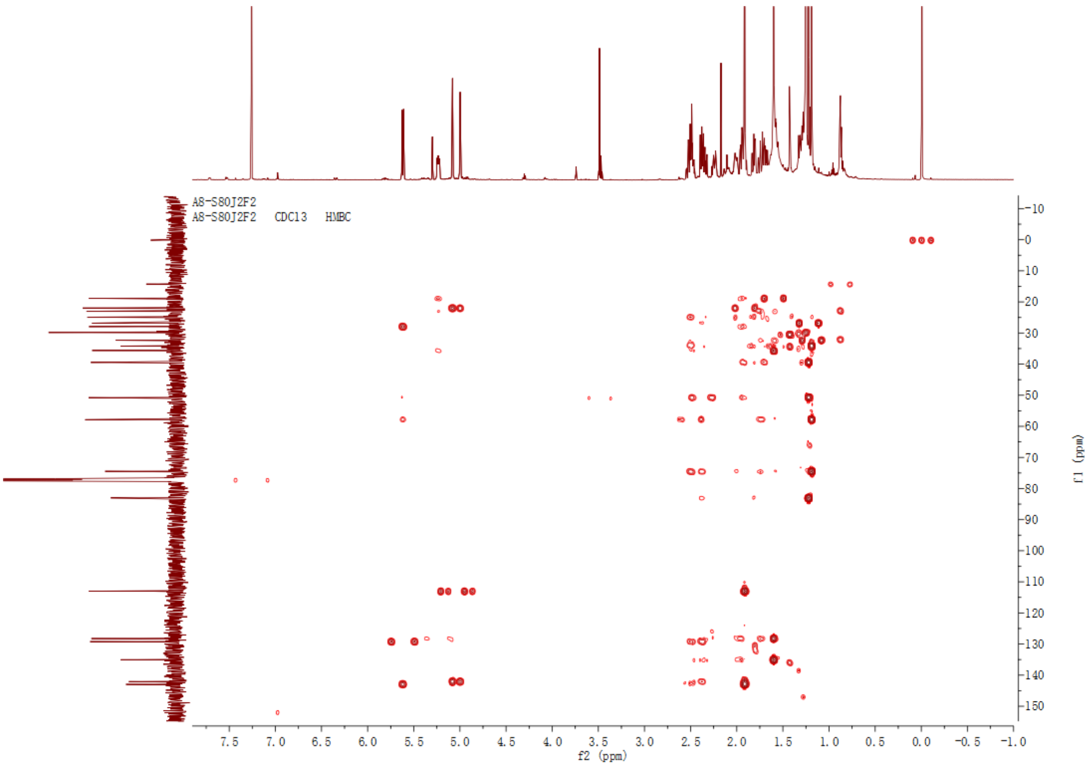

Figure S22. HMBC spectrum (600 MHz) of compound **3** in $CDCl_3$

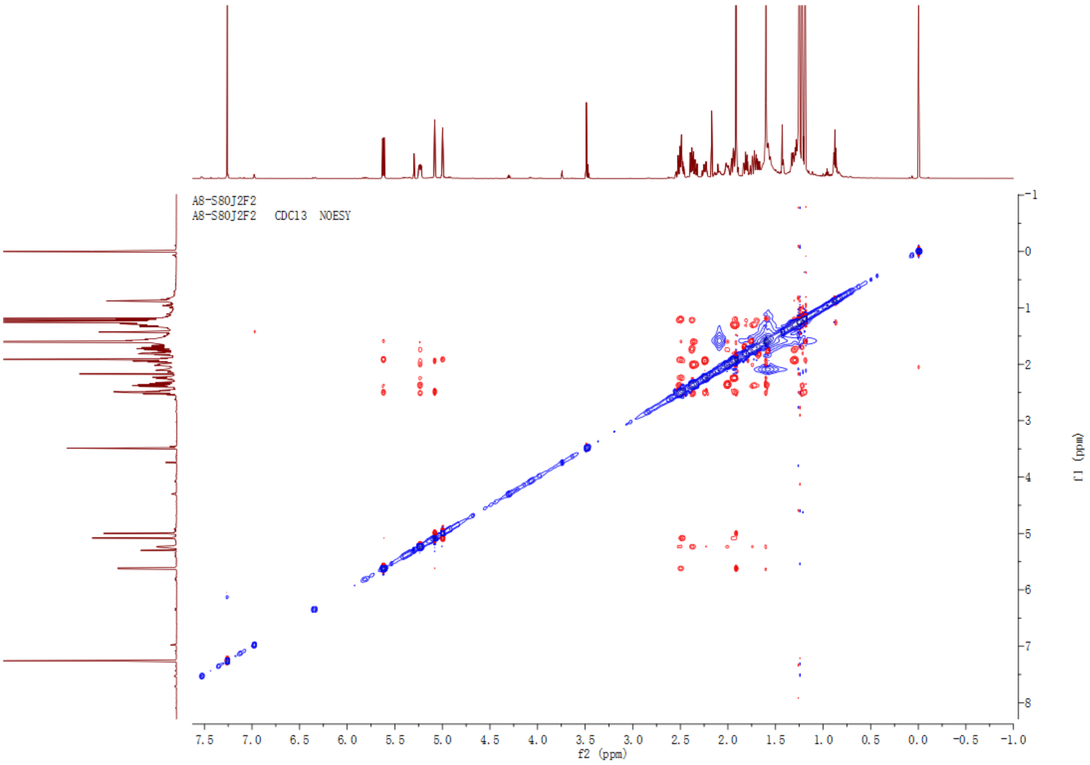

Figure S23. NOESY spectrum (600 MHz) of compound **3** in $CDCl_3$

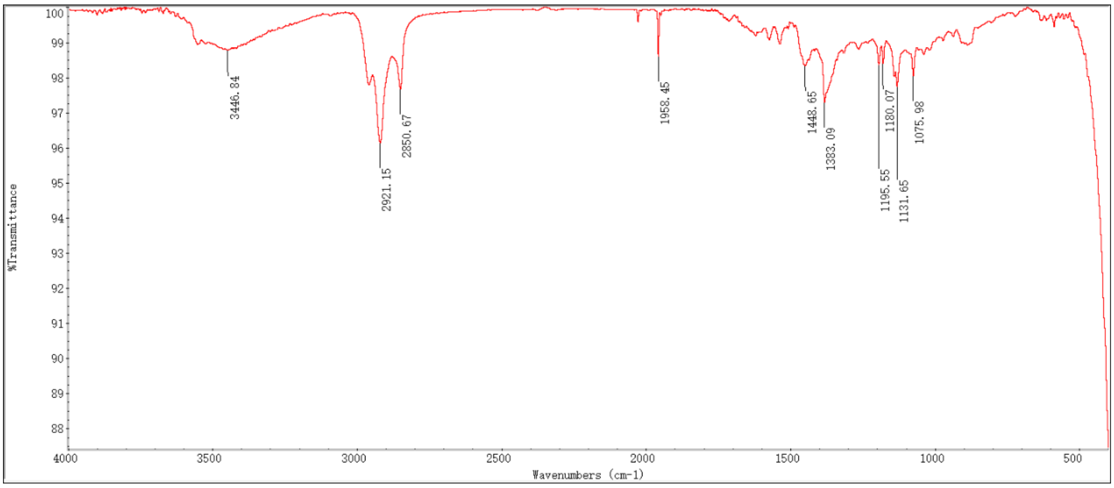

Figure S24. IR spectrum of compound **3**

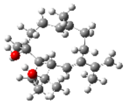

Conf. 1 14.70%

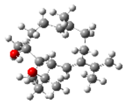

Conf. 2 19.61%

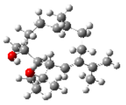

Conf. 3 17.21%

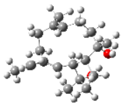

Conf. 4 1.58%

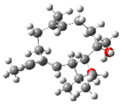

Conf. 5 2.41%

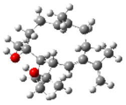

Conf. 6 0.23%

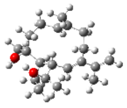

Conf. 7 4.62%

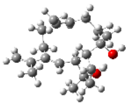

Conf. 8 5.00%

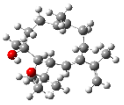

Conf. 9 0.29%

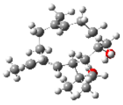

Conf. 10 1.74%

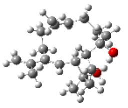

Conf. 11 5.20%

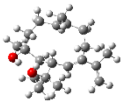

Conf. 12 0.34%

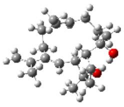

Conf. 13 8.06%

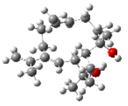

Conf. 14 2.57%

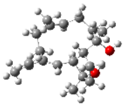

Conf. 15 0.50%

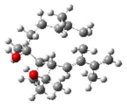

Conf. 16 14.70%

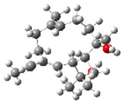

Conf. 17 0.47%

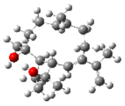

Conf. 18 0.10%

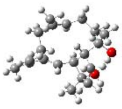

Conf. 19 0.62%

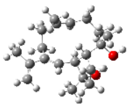

Conf. 20 0.05%

Figure S25. Re-optimized conformers above 1% population (OPLS_2005) of (3*S*,4*S*,7*R*,8*S*)-**2** calculated at the B3LYP/6-311G(d,p) level with IEFPCM solvent model for methanol.

Table S1. Cartesian coordinates for the re-optimized conformers of compound **2** at the B3LYP/6-311G(d,p) level with IEFPCM solvent model for methanol.

Compound **2**<br>Conformer 1		Standard orientation<br>(Ångstroms)		
I	atom	X	Y	Z
1	C	-0.35302500	-0.72289500	0.34886400
2	C	-1.39096700	-0.48022500	-0.81187400
3	C	-2.50172200	0.59446200	-0.61737000
4	C	0.98763100	-1.01277000	-0.28014200
5	C	-0.90971900	-1.96685900	1.12183400
6	C	-1.44421100	-2.85941400	-0.01657500
7	C	-1.99654700	-1.90099100	-1.09680700
8	C	-2.05137000	1.95637100	-0.03998800
9	C	-1.05053600	2.83112900	-0.83578900
10	C	0.39563700	2.42196400	-0.69004300
11	C	1.16070100	2.62251800	0.39061100
12	C	2.53172200	1.99087600	0.50047400
13	C	2.44745100	0.53858300	1.06525900
14	C	2.20761200	-0.50542600	-0.00744200
15	C	3.38501700	-0.95373000	-0.80264300
16	C	4.62894700	-0.49823600	-0.57451600
17	C	3.17912000	-1.96020200	-1.91626200
18	H	-0.29063800	0.11641200	1.03802700
19	C	0.09818700	-2.66834700	2.02517100
20	O	-1.96779400	-1.55541800	1.99895100
21	H	-0.83176500	-0.14329300	-1.68674500
22	O	-3.44423500	0.03753400	0.34537500
23	C	0.71005700	3.37676100	1.61760900
24	C	-3.25161100	0.79875300	-1.94353800
25	H	0.92903900	-1.74729700	-1.07755900
26	H	-2.20123000	-3.54901900	0.36398700
27	H	-0.62647600	-3.46369500	-0.41958500
28	H	-1.73770400	-2.24430000	-2.10020500
29	H	-3.08350600	-1.86080200	-1.04502300
30	H	-1.66399600	1.79042600	0.96774000
31	H	-2.97269300	2.54109000	0.08099300
32	H	-1.32698400	2.84796400	-1.89325300
33	H	-1.18113300	3.85889100	-0.48268200
34	H	0.82611400	1.85911800	-1.51443800
35	H	3.02494800	1.96787400	-0.47399400
36	H	3.16253700	2.59266700	1.16292200
37	H	3.37234100	0.30390500	1.59737900
38	H	1.65658200	0.49369700	1.81581700

39	H	5.46272200	-0.84650000	-1.17385300
40	H	4.86544700	0.22273900	0.19660300
41	H	2.48773100	-1.58461500	-2.67681000
42	H	4.12796700	-2.18494600	-2.40477300
43	H	2.76288700	-2.90008800	-1.54086200
44	H	-0.39570600	-3.49292000	2.54671500
45	H	0.48121400	-1.97394900	2.77781400
46	H	0.94188500	-3.06807100	1.46147500
47	H	-2.59270800	-1.00210700	1.49566700
48	H	-4.08794800	0.71644100	0.57867400
49	H	-0.28440900	3.81115800	1.51743500
50	H	0.70144100	2.72285700	2.49805500
51	H	1.41388400	4.18630200	1.84222600
52	H	-3.66652200	-0.14271000	-2.30585600
53	H	-4.07809300	1.50201600	-1.80432200
54	H	-2.59323400	1.19989800	-2.71718600

B3LYP/6-311G(d,p) Energy = -932.05466985 a.u.; Population = 14.70%

Compound **2**<br>Conformer 2		Standard orientation<br>(Ångstroms)		
I	atom	X	Y	Z
1	C	-0.36218900	-0.71243300	0.33918000
2	C	-1.40506000	-0.46571400	-0.81671100
3	C	-2.51865700	0.61949600	-0.61772700
4	C	0.97673800	-1.02035800	-0.28852900
5	C	-0.91315900	-1.94653400	1.10615900
6	C	-1.46466400	-2.84056600	-0.01557100
7	C	-2.02395100	-1.87977700	-1.08903100
8	C	-2.04230900	1.97928700	-0.04295800
9	C	-1.02071200	2.84474600	-0.82356000
10	C	0.41964400	2.41765600	-0.67521100
11	C	1.18853200	2.60933900	0.40447000
12	C	2.55275300	1.96212300	0.51216400
13	C	2.45341300	0.50689200	1.06645000
14	C	2.20106900	-0.52628700	-0.01361500
15	C	3.37244900	-0.98122000	-0.81495100
16	C	4.62144000	-0.54262100	-0.58310500
17	C	3.15318300	-1.97493200	-1.93718300
18	H	-0.28874800	0.12877000	1.02510400
19	C	0.07004800	-2.64119500	2.04242200
20	O	-2.00510900	-1.41805600	1.91278700
21	H	-0.84093600	-0.13901600	-1.69312000
22	O	-3.54691400	0.15342000	0.27707800

23	C	0.74821800	3.36864700	1.63220000
24	C	-3.23099600	0.84203000	-1.95987500
25	H	0.90861000	-1.74817600	-1.09098900
26	H	-2.21758800	-3.53699000	0.36479500
27	H	-0.64906700	-3.44772100	-0.41743400
28	H	-1.78825900	-2.23386800	-2.09435200
29	H	-3.10838900	-1.82094400	-1.01101400
30	H	-1.66634100	1.80968200	0.97001900
31	H	-2.96131100	2.56325800	0.07368100
32	H	-1.28918100	2.87446500	-1.88317100
33	H	-1.13767900	3.87249000	-0.46414000
34	H	0.84439400	1.84867900	-1.49869500
35	H	3.04774200	1.94101500	-0.46149600
36	H	3.18916100	2.55194400	1.17997600
37	H	3.37627000	0.25721800	1.59516300
38	H	1.66329600	0.46524400	1.81805400
39	H	5.45071400	-0.89511000	-1.18617400
40	H	4.86684200	0.16824700	0.19453400
41	H	2.46470900	-1.58476600	-2.69301400
42	H	4.09854700	-2.20583500	-2.42946800
43	H	2.72728500	-2.91350800	-1.56938500
44	H	-0.42537400	-3.46838100	2.56098600
45	H	0.43939900	-1.94083800	2.79542100
46	H	0.92379500	-3.04680600	1.49770000
47	H	-2.45987300	-2.16157400	2.32730500
48	H	-3.10303400	-0.32767700	0.99871800
49	H	-0.24328600	3.81006100	1.53382000
50	H	0.73665700	2.71575200	2.51348100
51	H	1.45901600	4.17294900	1.85436300
52	H	-3.63768800	-0.09534500	-2.34388300
53	H	-4.06220100	1.53880300	-1.82273000
54	H	-2.55604000	1.25358400	-2.71307700

B3LYP/6-311G(d,p) Energy =-932.05494169 a.u.; Population = 19.61%

Compound **2**<br>Conformer 3		Standard orientation<br>(Ångstroms)		
I	atom	X	Y	Z
1	C	-0.32958300	-0.70095900	0.34023900
2	C	-1.36121700	-0.49735200	-0.83096200
3	C	-2.52601900	0.53870500	-0.65760900
4	C	1.01307900	-1.02423000	-0.27028300
5	C	-0.89561700	-1.90517600	1.15227700
6	C	-1.46010100	-2.83732300	0.07189700

7	C	-1.90900900	-1.93812500	-1.10646500
8	C	-2.12310000	1.90513200	-0.04391600
9	C	-1.12986100	2.83252300	-0.78937500
10	C	0.32276900	2.43698800	-0.67953700
11	C	1.11409500	2.62960100	0.38348900
12	C	2.49807600	2.01933600	0.44524100
13	C	2.46170900	0.57387500	1.03228000
14	C	2.22860500	-0.49749600	-0.01577200
15	C	3.40695900	-0.95245000	-0.80606400
16	C	4.64276200	-0.46235800	-0.60897800
17	C	3.20853100	-2.00047500	-1.88189200
18	H	-0.25375600	0.17183100	0.98617300
19	C	0.08240500	-2.57875200	2.10885900
20	O	-2.03929500	-1.44199300	1.93014200
21	H	-0.79720000	-0.14816900	-1.69842300
22	O	-3.57009000	0.01379000	0.18437600
23	C	0.68554600	3.35583200	1.63526200
24	C	-3.19451200	0.75677500	-2.02274800
25	H	0.95515100	-1.78505000	-1.04235400
26	H	-2.27428100	-3.43793000	0.48312300
27	H	-0.67526300	-3.52960400	-0.24165700
28	H	-1.53187400	-2.32707700	-2.05439400
29	H	-2.99520800	-1.91527700	-1.17580000
30	H	-1.75702300	1.73273400	0.97265400
31	H	-3.06889400	2.44585700	0.06709400
32	H	-1.41164900	2.90557900	-1.84342800
33	H	-1.26981800	3.83799500	-0.37946600
34	H	0.74054700	1.89216800	-1.52301900
35	H	2.95162700	1.99127500	-0.54820300
36	H	3.14574500	2.63976600	1.07364500
37	H	3.40149100	0.36962400	1.55010700
38	H	1.68787100	0.52151800	1.80004100
39	H	5.47702200	-0.81285100	-1.20627100
40	H	4.87184200	0.29118500	0.13263800
41	H	4.15739700	-2.22923500	-2.36843400
42	H	2.80748600	-2.93200800	-1.47088800
43	H	2.50768200	-1.66111900	-2.65073800
44	H	-0.41987200	-3.39364100	2.63614400
45	H	0.45395200	-1.86753700	2.85362500
46	H	0.94366600	-2.98464500	1.57675700
47	H	-1.70518100	-0.89512500	2.65202400
48	H	-3.13888100	-0.44983100	0.92533900
49	H	-0.32520900	3.75860800	1.57504600

50	H	0.72840300	2.69238600	2.50750400
51	H	1.37126200	4.18475100	1.84564300
52	H	-3.55326100	-0.18875700	-2.43371100
53	H	-4.05447100	1.42195700	-1.90763000
54	H	-2.50732700	1.20180400	-2.74494200

B3LYP/6-311G(d,p) Energy =-932.05481857 a.u.; Population = 17.21%

Compound **2**<br>Conformer 4		Standard orientation<br>(Ångstroms)		
I	atom	X	Y	Z
1	C	0.40540100	-0.78860400	-0.30118300
2	C	1.45397000	-0.41419300	0.81636800
3	C	2.50234500	0.69634200	0.51447200
4	C	-0.92390600	-1.03438800	0.37080900
5	C	0.97437300	-2.09370300	-0.95264800
6	C	1.53594400	-2.85517000	0.26389700
7	C	2.13971100	-1.77727900	1.18796700
8	C	1.96205000	2.00883600	-0.09938000
9	C	0.94794600	2.86793800	0.69846200
10	C	-0.48760900	2.41498000	0.57434900
11	C	-1.25991800	2.55571300	-0.51070800
12	C	-2.60757400	1.87157900	-0.60263500
13	C	-2.45412900	0.38966700	-1.06314700
14	C	-2.14303100	-0.54300000	0.08969200
15	C	-3.29196600	-0.90037300	0.97962100
16	C	-3.19173200	-0.83655000	2.31380400
17	C	-4.58477200	-1.33661400	0.32401500
18	H	0.32010100	-0.02068500	-1.06873100
19	C	-0.02876400	-2.90122700	-1.76799900
20	O	2.02039500	-1.75948200	-1.87691700
21	H	0.89718500	-0.04412600	1.67951800
22	O	3.41626800	0.13310700	-0.47160700
23	C	-0.83412300	3.28211500	-1.76342200
24	C	3.31038100	0.99958900	1.78684000
25	H	-0.86808600	-1.71249900	1.22005600
26	H	2.26936100	-3.60031700	-0.05314900
27	H	0.72395900	-3.38806100	0.76793400
28	H	1.99357900	-2.02212600	2.24162400
29	H	3.21409100	-1.70713500	1.02231100
30	H	1.54526600	1.78265200	-1.08329100
31	H	2.84809600	2.63075100	-0.28179600
32	H	1.23541700	2.90749200	1.75233100
33	H	1.04463100	3.89433500	0.33061600

34	H	-0.89947000	1.86864300	1.41857600
35	H	-3.12364200	1.89336400	0.36141100
36	H	-3.24433000	2.40188700	-1.31748800
37	H	-3.38313100	0.07045200	-1.54294600
38	H	-1.67944700	0.32678100	-1.82980300
39	H	-4.01442600	-1.13351400	2.95649300
40	H	-2.28927300	-0.48428100	2.80002300
41	H	-5.28905000	-1.71159500	1.06867400
42	H	-5.06905400	-0.50933800	-0.20433900
43	H	-4.40816400	-2.12514500	-0.41475300
44	H	0.47665600	-3.76441300	-2.21002200
45	H	-0.43426300	-2.29404800	-2.58191200
46	H	-0.85705000	-3.25917100	-1.15541200
47	H	2.60641900	-1.10133300	-1.46190500
48	H	4.00879400	0.82793900	-0.78084800
49	H	0.14224700	3.75842800	-1.67633400
50	H	-0.79683800	2.59780000	-2.61975200
51	H	-1.56780700	4.05519000	-2.01882300
52	H	3.79073200	0.09836800	2.16946300
53	H	4.09185200	1.73352500	1.56937600
54	H	2.67477300	1.40724200	2.57605400

B3LYP/6-311G(d,p) Energy =-932.05256389 a.u.; Population = 1.58%

Compound **2**<br>Conformer 5		Standard orientation<br>(Ångstroms)		
I	atom	X	Y	Z
1	C	0.39905300	-0.77138900	-0.28940600
2	C	1.45156300	-0.41216700	0.82872800
3	C	2.52415200	0.68995100	0.53261200
4	C	-0.92802400	-1.03801600	0.38144100
5	C	0.96183500	-2.05501600	-0.95841700
6	C	1.54280300	-2.84186500	0.22638700
7	C	2.11980500	-1.78296500	1.19035000
8	C	1.98998800	2.00193200	-0.10025800
9	C	0.96127600	2.87770700	0.66057200
10	C	-0.47279000	2.41865900	0.54659200
11	C	-1.25390900	2.54485700	-0.53396400
12	C	-2.60504200	1.86423200	-0.60416300
13	C	-2.46536000	0.37783600	-1.05307600
14	C	-2.14832300	-0.54890600	0.10322900
15	C	-3.29279600	-0.90433100	0.99977600
16	C	-3.18568000	-0.83021700	2.33277300
17	C	-4.58705800	-1.34874900	0.35294500

18	H	0.30363700	0.01000000	-1.04169400
19	C	-0.01988800	-2.84310000	-1.81902300
20	O	2.03520100	-1.57822400	-1.82011000
21	H	0.89128500	-0.04236100	1.69038100
22	O	3.53607500	0.20203000	-0.36978100
23	C	-0.83680800	3.25187100	-1.80075300
24	C	3.27322400	1.01240400	1.83459400
25	H	-0.86556700	-1.71474600	1.23120700
26	H	2.29063000	-3.56869400	-0.10458700
27	H	0.73843000	-3.41040400	0.70134600
28	H	1.93941200	-2.05616200	2.23167000
29	H	3.19777400	-1.70545600	1.05677900
30	H	1.59528600	1.76921100	-1.09325300
31	H	2.88707400	2.60590500	-0.27184900
32	H	1.24125800	2.95246500	1.71490700
33	H	1.05399500	3.89343300	0.26212100
34	H	-0.88043500	1.88319900	1.40024700
35	H	-3.11017000	1.89750100	0.36537400
36	H	-3.24804000	2.39045000	-1.31655300
37	H	-3.40122100	0.05922800	-1.51951500
38	H	-1.70005400	0.30357100	-1.82819300
39	H	-4.00472000	-1.12316100	2.98188200
40	H	-2.28162900	-0.47197200	2.81152700
41	H	-5.28628000	-1.72135500	1.10351900
42	H	-5.07672700	-0.52641200	-0.17805000
43	H	-4.41150900	-2.14149700	-0.38150300
44	H	0.48323600	-3.70526800	-2.26864400
45	H	-0.40783000	-2.21690700	-2.62622200
46	H	-0.86121000	-3.21209400	-1.23047100
47	H	2.51032700	-2.34535000	-2.16223900
48	H	3.09010800	-0.36041900	-1.02884600
49	H	0.14715500	3.71515800	-1.73183000
50	H	-0.81986600	2.55749800	-2.64965500
51	H	-1.56368800	4.03173200	-2.05575000
52	H	3.72110900	0.11261500	2.26013400
53	H	4.07807400	1.72240100	1.62659200
54	H	2.61183700	1.45106600	2.58466200

B3LYP/6-311G(d,p) Energy =-932.05296235 a.u.; Population = 2.41%

Compound **2**<br>Conformer 6		Standard orientation<br>(Ångstroms)		
I	atom	X	Y	Z
1	C	-0.29423800	-0.71082600	0.27699700

2	C	-1.32040000	-0.49481100	-0.89280300
3	C	-2.54397800	0.44749100	-0.67505600
4	C	1.06066800	-0.97411400	-0.33581000
5	C	-0.83899500	-1.95608300	1.05445900
6	C	-1.38808500	-2.86623000	-0.07134900
7	C	-1.75370200	-1.95081300	-1.26912600
8	C	-2.25758200	1.77482900	0.06366800
9	C	-1.33673200	2.82910500	-0.59813100
10	C	0.13794900	2.50863100	-0.55702300
11	C	0.93734500	2.61662100	0.51130500
12	C	2.36464400	2.11916200	0.46834300
13	C	2.50390800	0.65284600	0.98462800
14	C	2.25773500	-0.42812600	-0.05761400
15	C	3.46643100	-0.92099300	-0.79105200
16	C	3.72005200	-2.23052300	-0.91132900
17	C	4.41399000	0.10098700	-1.38021200
18	H	-0.24902700	0.13298600	0.96088100
19	C	0.18114400	-2.64943000	1.95102900
20	O	-1.88409700	-1.54120200	1.94761400
21	H	-0.77875600	-0.04870600	-1.72891800
22	O	-3.49137200	-0.28776900	0.15615500
23	C	0.49023900	3.15174700	1.84967400
24	C	-3.22552500	0.71832000	-2.02526900
25	H	1.05285000	-1.72342100	-1.12537700
26	H	-2.24440600	-3.43188500	0.30249400
27	H	-0.62563000	-3.59268200	-0.36514800
28	H	-1.23067300	-2.27520400	-2.17105900
29	H	-2.81896600	-1.99718400	-1.48814900
30	H	-1.88237500	1.53722900	1.06192800
31	H	-3.23649200	2.24914400	0.21180700
32	H	-1.64838700	2.99455800	-1.63281900
33	H	-1.52364200	3.77564600	-0.08130400
34	H	0.57169200	2.10510900	-1.46884000
35	H	2.75555500	2.18764500	-0.54834500
36	H	2.99323100	2.76206800	1.09446900
37	H	3.51364400	0.51996000	1.38439700
38	H	1.82823400	0.50864300	1.83115800
39	H	4.58833600	-2.58791100	-1.45577500
40	H	3.07537600	-2.97935700	-0.46603800
41	H	4.80916500	0.77672100	-0.61571300
42	H	5.26051700	-0.38833100	-1.86521100
43	H	3.90646300	0.72546500	-2.12289400
44	H	-0.30192200	-3.47999800	2.47326800

45	H	0.56128500	-1.95250600	2.70295400
46	H	1.02596400	-3.03962100	1.38259300
47	H	-2.57769900	-1.10628000	1.41821600
48	H	-4.23280000	0.29344600	0.36324100
49	H	-0.54572500	3.48910700	1.85352300
50	H	0.59486000	2.39182100	2.63298200
51	H	1.12372500	3.99375600	2.15155900
52	H	-3.52582000	-0.21512300	-2.50331800
53	H	-4.12234700	1.32826300	-1.87980200
54	H	-2.56169200	1.25165800	-2.70888800

B3LYP/6-311G(d,p) Energy =-932.05074915 a.u.; Population = 0.23%

Compound **2**<br>Conformer 7		Standard orientation<br>(Ångstroms)		
I	atom	X	Y	Z
1	C	-0.30103800	-0.69375400	0.34567300
2	C	-1.31271800	-0.50574900	-0.84252800
3	C	-2.51113200	0.48344900	-0.66522800
4	C	1.04593300	-1.02296800	-0.24787000
5	C	-0.89185600	-1.87360900	1.18971200
6	C	-1.50121900	-2.81688400	0.12131200
7	C	-1.77230400	-1.96789800	-1.14820300
8	C	-2.16907500	1.84802600	-0.02893400
9	C	-1.21177800	2.80654500	-0.77909200
10	C	0.25289500	2.44827400	-0.69700000
11	C	1.05474900	2.64844600	0.35632600
12	C	2.45309600	2.07055700	0.39180300
13	C	2.46367800	0.63754800	1.01037400
14	C	2.25220500	-0.46792600	-0.00732200
15	C	3.44069800	-0.92597000	-0.77967600
16	C	4.66284200	-0.39205200	-0.61178800
17	C	3.26917400	-2.02744400	-1.80589600
18	H	-0.23576400	0.18507100	0.98127300
19	C	0.11131400	-2.56822200	2.10410300
20	O	-1.90206200	-1.36444400	2.07404200
21	H	-0.75554400	-0.11776000	-1.69787800
22	O	-3.49223400	-0.06527500	0.27037700
23	C	0.62708900	3.35030400	1.62209500
24	C	-3.21471200	0.69042500	-2.01416900
25	H	1.00619400	-1.81185500	-0.99253800
26	H	-2.40727800	-3.27630500	0.52223900
27	H	-0.80467000	-3.62821600	-0.10465800
28	H	-1.20577700	-2.36136000	-1.99441100

29	H	-2.81980300	-2.01954300	-1.44962800
30	H	-1.79049500	1.67506000	0.98174500
31	H	-3.13107900	2.35613000	0.09033800
32	H	-1.51314100	2.88558200	-1.82722900
33	H	-1.37365800	3.80166200	-0.35364300
34	H	0.67129800	1.92464500	-1.55339100
35	H	2.87959000	2.03140200	-0.61324100
36	H	3.10303600	2.71878500	0.98930500
37	H	3.41331500	0.47281300	1.52407000
38	H	1.69852300	0.58005500	1.78622400
39	H	5.50466400	-0.74540300	-1.19670000
40	H	4.87253900	0.40166900	0.09281400
41	H	2.56300300	-1.74083800	-2.59123300
42	H	4.22425600	-2.25685300	-2.27990100
43	H	2.88811400	-2.94758600	-1.35246200
44	H	-0.39543800	-3.35801500	2.66553600
45	H	0.52765200	-1.85593100	2.82145500
46	H	0.93263100	-3.01279100	1.54089200
47	H	-2.56867100	-0.90096400	1.53352200
48	H	-4.02741900	-0.72040100	-0.19204800
49	H	-0.39374600	3.72934700	1.58123500
50	H	0.69839800	2.67912000	2.48627100
51	H	1.29575600	4.19330500	1.83108700
52	H	-3.58755700	-0.25354900	-2.42004400
53	H	-4.06461900	1.36610500	-1.89138100
54	H	-2.53699800	1.11779000	-2.75512200

B3LYP/6-311G(d,p) Energy =-932.05357833 a.u.; Population = 4.62%

Compound **2**<br>Conformer 8		Standard orientation<br>(Ångstroms)		
I	atom	X	Y	Z
1	C	0.38164900	-0.74031500	-0.35223800
2	C	1.28891800	-0.16846300	0.79566600
3	C	2.51566200	0.72829400	0.43742400
4	C	-0.99904800	-0.96861400	0.21445300
5	C	1.06709000	-2.08867800	-0.76003400
6	C	1.59786700	-2.64641200	0.58841500
7	C	1.70780400	-1.45480000	1.57624600
8	C	2.30267100	1.79821000	-0.66067800
9	C	1.25184400	2.91517300	-0.43088200
10	C	-0.10674400	2.55493200	-0.96027700
11	C	-1.27474500	2.44530000	-0.31992300
12	C	-2.48092900	1.90563600	-1.07662800

13	C	-2.34372800	0.39663600	-1.41291700
14	C	-2.19731900	-0.50681600	-0.20249800
15	C	-3.43072200	-0.87386000	0.54665100
16	C	-4.65633400	-0.48555100	0.15456700
17	C	-3.30576700	-1.72034900	1.79671000
18	H	0.34418600	-0.08808200	-1.22067500
19	C	0.16226600	-3.06434700	-1.50503100
20	O	2.14156600	-1.82003700	-1.67360800
21	H	0.66436000	0.46295200	1.43098000
22	O	3.55461800	-0.16691300	-0.06103000
23	C	-1.49741300	2.75736100	1.13667600
24	C	3.05073400	1.38610500	1.71985500
25	H	-0.99745300	-1.60372900	1.09493300
26	H	2.55902000	-3.13632400	0.41844100
27	H	0.91596200	-3.40710800	0.97766100
28	H	1.03602800	-1.60288200	2.42444100
29	H	2.71371400	-1.36776100	1.98303200
30	H	2.09029300	1.28609600	-1.60359200
31	H	3.28017200	2.27743600	-0.79313700
32	H	1.21543200	3.20070400	0.62175300
33	H	1.60926100	3.80202200	-0.96843200
34	H	-0.10311600	2.30567600	-2.02259000
35	H	-3.39090200	2.07292800	-0.49507400
36	H	-2.60636200	2.44926100	-2.01892900
37	H	-3.21053000	0.08757600	-2.00445300
38	H	-1.47945700	0.27241900	-2.06619100
39	H	-5.53512200	-0.77252600	0.72111900
40	H	-4.83255700	0.11806600	-0.72578400
41	H	-2.85807700	-2.69539100	1.58127100
42	H	-2.67510500	-1.23774400	2.54962800
43	H	-4.28773900	-1.89275900	2.23902400
44	H	0.73324600	-3.95946900	-1.76662000
45	H	-0.20270500	-2.61202800	-2.43130700
46	H	-0.69734800	-3.36335400	-0.90392600
47	H	2.78776200	-1.25558600	-1.21065300
48	H	4.31353900	0.36504200	-0.32874200
49	H	-0.59879000	3.10699000	1.64580300
50	H	-2.26677200	3.53114100	1.24477600
51	H	-1.86921600	1.87290600	1.66523900
52	H	3.29570400	0.63481200	2.47186200
53	H	3.95965200	1.95452500	1.50103400
54	H	2.32091800	2.07312700	2.15286200

B3LYP/6-311G(d,p) Energy =-932.05365298 a.u.; Population = 5.00%

Compound **2**<br>Conformer 9		Standard orientation<br>(Ångstroms)		
I	atom	X	Y	Z
1	C	-0.33784700	-0.71301100	0.27160900
2	C	-1.38656400	-0.46662900	-0.87614000
3	C	-2.56327900	0.54263100	-0.63827700
4	C	1.01151800	-0.97087500	-0.35993800
5	C	-0.85230000	-1.98404600	1.00293800
6	C	-1.36910300	-2.86692800	-0.14520300
7	C	-1.92164700	-1.89961600	-1.21909500
8	C	-2.17610000	1.88141200	0.04053100
9	C	-1.19520300	2.85640300	-0.65617600
10	C	0.26397400	2.48249300	-0.55873800
11	C	1.03585400	2.59646500	0.52938000
12	C	2.44404000	2.04444600	0.54272900
13	C	2.50084800	0.56003700	1.02036000
14	C	2.22324200	-0.47094300	-0.06178600
15	C	3.41487000	-0.97350200	-0.81795500
16	C	3.61827700	-2.28568900	-0.99304500
17	C	4.40493900	0.03277900	-1.36219100
18	H	-0.28389000	0.11003400	0.98112900
19	C	0.14690200	-2.67217600	1.92732700
20	O	-1.96408500	-1.51511900	1.82041200
21	H	-0.83630900	-0.06730200	-1.73080700
22	O	-3.58717800	-0.03632200	0.19336800
23	C	0.57038700	3.18670100	1.83807600
24	C	-3.25420300	0.81555900	-1.98184500
25	H	0.98054800	-1.67911100	-1.18544400
26	H	-2.11910000	-3.57932800	0.21046600
27	H	-0.53735500	-3.45616000	-0.54022400
28	H	-1.61337800	-2.21113400	-2.21897600
29	H	-3.01022400	-1.89704800	-1.20086400
30	H	-1.80882900	1.66020200	1.04675000
31	H	-3.12823400	2.40451700	0.17882500
32	H	-1.47316100	2.97412600	-1.70717200
33	H	-1.35212300	3.84021500	-0.20120000
34	H	0.70999100	2.03254600	-1.44239000
35	H	2.88866500	2.12537100	-0.45043300
36	H	3.06570000	2.64301400	1.21728200
37	H	3.49480900	0.36533000	1.43477200
38	H	1.80024400	0.42762000	1.84833500

39	H	4.47393200	-2.65254300	-1.55094500
40	H	2.94483700	-3.02781100	-0.58041300
41	H	5.22998800	-0.47127200	-1.86851500
42	H	3.92615600	0.71016700	-2.07683600
43	H	4.82825900	0.65633300	-0.56902000
44	H	-0.32724200	-3.52578200	2.42283400
45	H	0.49144300	-1.97993400	2.69937300
46	H	1.01514900	-3.04040000	1.37938500
47	H	-2.37700000	-2.28434600	2.23178600
48	H	-3.13145300	-0.51071200	0.91236700
49	H	-0.44914200	3.56891100	1.79795600
50	H	0.61566300	2.44324700	2.64314800
51	H	1.23072600	4.00775600	2.14037500
52	H	-3.60140000	-0.11516900	-2.43467100
53	H	-4.12420800	1.45839000	-1.82313300
54	H	-2.58458600	1.30872900	-2.68936300

B3LYP/6-311G(d,p) Energy =-932.05097040 a.u.; Population = 0.29%

Compound **2**<br>Conformer 10		Standard orientation<br>(Ångstroms)		
I	atom	X	Y	Z
1	C	0.36384700	-0.75819800	-0.28466200
2	C	1.41965300	-0.45092400	0.84296900
3	C	2.54403000	0.60513800	0.57036600
4	C	-0.96520000	-1.02822800	0.38112300
5	C	0.92117100	-2.02108600	-1.00602500
6	C	1.51088300	-2.85659300	0.13634200
7	C	2.02084600	-1.85336500	1.19755700
8	C	2.08165900	1.92801500	-0.09490300
9	C	1.07519700	2.85806000	0.62995600
10	C	-0.37098200	2.43244000	0.54468700
11	C	-1.17131800	2.56864100	-0.52033100
12	C	-2.54566300	1.93250300	-0.55139500
13	C	-2.47481100	0.44752400	-1.02176800
14	C	-2.17708100	-0.51145300	0.11449100
15	C	-3.32834000	-0.86738800	1.00159200
16	C	-3.22192300	-0.82002000	2.33590800
17	C	-4.62767100	-1.28218500	0.34517800
18	H	0.26760300	0.05828700	-0.99946400
19	C	-0.06941700	-2.77576700	-1.88580500
20	O	2.04897800	-1.61789400	-1.83934000
21	H	0.86792900	-0.06347800	1.70207700
22	O	3.56961600	0.06659300	-0.28696200

23	C	-0.76029900	3.24619800	-1.80503300
24	C	3.25909400	0.91448100	1.89452600
25	H	-0.91274200	-1.72856000	1.21244300
26	H	2.29688000	-3.51445200	-0.24098300
27	H	0.72422000	-3.49418800	0.54652300
28	H	1.72872600	-2.16677700	2.20169000
29	H	3.10829000	-1.80374200	1.17814200
30	H	1.70151000	1.69799700	-1.09452000
31	H	3.00674600	2.49246700	-0.25245300
32	H	1.36292500	2.96926800	1.67905500
33	H	1.19154600	3.85326000	0.18879000
34	H	-0.77566300	1.91686800	1.41205100
35	H	-3.01562700	1.97120700	0.43539300
36	H	-3.19425800	2.48944200	-1.23504300
37	H	-3.43027200	0.17526200	-1.47714500
38	H	-1.72731600	0.35385900	-1.81206300
39	H	-4.04586800	-1.11363500	2.97850500
40	H	-2.31362400	-0.48340900	2.82225900
41	H	-5.33157800	-1.66229600	1.08753500
42	H	-5.10756000	-0.44298800	-0.16803500
43	H	-4.46137500	-2.06108800	-0.40603800
44	H	0.42941700	-3.62657200	-2.35679600
45	H	-0.46205600	-2.12900700	-2.67726000
46	H	-0.91641600	-3.14469300	-1.30581000
47	H	1.69677200	-1.14208800	-2.60185300
48	H	3.12865100	-0.48160600	-0.96139000
49	H	0.24396400	3.66758000	-1.76743700
50	H	-0.79572100	2.54370000	-2.64660300
51	H	-1.46031100	4.05366900	-2.04904800
52	H	3.65699300	0.00283000	2.34406000
53	H	4.09762300	1.59072400	1.70809300
54	H	2.58979400	1.38581100	2.61710500

B3LYP/6-311G(d,p) Energy = -932.05265893 a.u.; Population = 1.74%

Compound **2**<br>Conformer 11		Standard orientation<br>(Ångstroms)		
I	atom	X	Y	Z
1	C	0.40332900	-0.73401300	-0.34647200
2	C	1.31445600	-0.15276500	0.79556100
3	C	2.53347900	0.76734000	0.42697600
4	C	-0.97682100	-0.96969900	0.22312300
5	C	1.06900000	-2.08436600	-0.73076000
6	C	1.57715700	-2.64218800	0.61685700

7	C	1.77730100	-1.43108700	1.56619100
8	C	2.27760500	1.84902100	-0.65727400
9	C	1.20090000	2.94040200	-0.42094400
10	C	-0.14665600	2.56185500	-0.96479200
11	C	-1.31896700	2.42671400	-0.33703400
12	C	-2.50563900	1.86424900	-1.10694000
13	C	-2.34094300	0.35317100	-1.42266200
14	C	-2.18027200	-0.52841500	-0.19878500
15	C	-3.40671800	-0.89605000	0.56249400
16	C	-4.63862500	-0.54112500	0.15970300
17	C	-3.26611700	-1.70552000	1.83506900
18	H	0.36311200	-0.08966000	-1.22117600
19	C	0.18885100	-3.05364200	-1.51450300
20	O	2.19082800	-1.71546100	-1.58614500
21	H	0.67677200	0.45785300	1.43888200
22	O	3.64854300	-0.01163500	-0.04390500
23	C	-1.56100700	2.72787800	1.11902700
24	C	3.04345100	1.43622200	1.71193000
25	H	-0.96511300	-1.58693800	1.11601300
26	H	2.49714100	-3.21321300	0.46629000
27	H	0.83911200	-3.33797800	1.02437400
28	H	1.18912300	-1.56308300	2.47671600
29	H	2.81921200	-1.34123000	1.86638700
30	H	2.07420900	1.34276200	-1.60691000
31	H	3.24752800	2.33864900	-0.78570800
32	H	1.14918500	3.21037400	0.63549600
33	H	1.54228600	3.84353100	-0.94266200
34	H	-0.12936300	2.31914000	-2.02880800
35	H	-3.42765400	2.02586700	-0.54293400
36	H	-2.62216600	2.39383100	-2.05823700
37	H	-3.20033900	0.01919500	-2.01142300
38	H	-1.47285000	0.23643100	-2.07222000
39	H	-5.51232300	-0.82816900	0.73398500
40	H	-4.82540600	0.03377800	-0.73744100
41	H	-2.80522900	-2.67979700	1.64544400
42	H	-2.64053200	-1.19317700	2.57240700
43	H	-4.24433700	-1.87994000	2.28477200
44	H	0.75425800	-3.95775000	-1.76397600
45	H	-0.14889100	-2.59399700	-2.44656900
46	H	-0.68724000	-3.35468100	-0.93847200
47	H	2.65048100	-2.52565500	-1.83881700
48	H	3.29522200	-0.61247500	-0.72489600
49	H	-0.67066000	3.08404800	1.63800300

50	H	-2.33951200	3.49312400	1.22353900
51	H	-1.92823500	1.83711500	1.64003000
52	H	3.29138400	0.68930100	2.46857100
53	H	3.95014300	2.00548400	1.49145300
54	H	2.30312200	2.11614500	2.13770700

B3LYP/6-311G(d,p) Energy =-932.05368953 a.u.; Population = 5.20%

Compound **2**<br>Conformer 12		Standard orientation<br>(Ångstroms)		
I	atom	X	Y	Z
1	C	-0.29416600	-0.70352000	0.26884500
2	C	-1.31955200	-0.49088400	-0.90224200
3	C	-2.56268600	0.44175300	-0.68341400
4	C	1.06325000	-0.98228100	-0.33439900
5	C	-0.84056800	-1.94180400	1.04321600
6	C	-1.40730500	-2.85145800	-0.06084300
7	C	-1.74793300	-1.94615200	-1.27336900
8	C	-2.27685300	1.76972900	0.06469700
9	C	-1.35083900	2.83564300	-0.57206500
10	C	0.12278700	2.51114500	-0.54594000
11	C	0.93690800	2.61638900	0.51171000
12	C	2.36297400	2.11666900	0.44918900
13	C	2.51047000	0.65382800	0.97330700
14	C	2.26022100	-0.43494600	-0.05987100
15	C	3.46732400	-0.93405600	-0.79207500
16	C	3.72432900	-2.24420200	-0.89425900
17	C	4.40695900	0.08374700	-1.40026300
18	H	-0.23814700	0.15205300	0.93943000
19	C	0.15249400	-2.63379700	1.97151800
20	O	-1.98243700	-1.52089700	1.84892700
21	H	-0.77092300	-0.04572400	-1.73503900
22	O	-3.59393200	-0.22995500	0.06487700
23	C	0.50956000	3.15322100	1.85605300
24	C	-3.20091600	0.73701300	-2.04788500
25	H	1.05309000	-1.73777100	-1.11756200
26	H	-2.27665000	-3.39030800	0.32154800
27	H	-0.65840900	-3.59907300	-0.33098900
28	H	-1.21282900	-2.28657300	-2.16235500
29	H	-2.81112400	-1.98648000	-1.50200400
30	H	-1.90925700	1.52976100	1.06702400
31	H	-3.26223000	2.22521800	0.20814400
32	H	-1.66439100	3.02703700	-1.60200600
33	H	-1.53115200	3.77119600	-0.03270500

34	H	0.54516200	2.10545200	-1.46243400
35	H	2.73748000	2.17787200	-0.57414500
36	H	3.00243200	2.76294200	1.06070800
37	H	3.52352600	0.52523400	1.36568600
38	H	1.84254300	0.51342700	1.82674800
39	H	4.59171200	-2.60682900	-1.43659200
40	H	3.08361800	-2.98846200	-0.43574700
41	H	4.80487200	0.77040900	-0.64694700
42	H	5.25188400	-0.40870800	-1.88481000
43	H	3.89202700	0.69697100	-2.14716500
44	H	-0.34076700	-3.46352200	2.48418900
45	H	0.52954000	-1.93914700	2.72912500
46	H	1.00941300	-3.02400800	1.42107600
47	H	-1.65299600	-0.97559100	2.57399400
48	H	-3.16188100	-0.64666600	0.83267900
49	H	-0.53017400	3.47802600	1.87739400
50	H	0.63794800	2.39881400	2.64126200
51	H	1.13869600	4.00409000	2.14232000
52	H	-3.48011500	-0.18941500	-2.55337000
53	H	-4.10801000	1.33110400	-1.90812100
54	H	-2.52266500	1.28927600	-2.70122000

B3LYP/6-311G(d,p) Energy = -932.05112692 a.u.; Population = 0.34%

Compound **2**<br>Conformer 13		Standard orientation<br>(Ångstroms)		
I	atom	X	Y	Z
1	C	0.38108800	-0.73279900	-0.33903800
2	C	1.28917000	-0.15608800	0.80616900
3	C	2.53543700	0.72608500	0.43931800
4	C	-1.00136400	-0.97165500	0.22171600
5	C	1.06484500	-2.07791000	-0.73810200
6	C	1.63749900	-2.62258800	0.58756100
7	C	1.70418700	-1.43879800	1.58751300
8	C	2.31232400	1.80076200	-0.65891600
9	C	1.25538500	2.91483500	-0.43964200
10	C	-0.10362200	2.55137800	-0.96577200
11	C	-1.27147000	2.44197000	-0.32451500
12	C	-2.47900500	1.90009000	-1.07772400
13	C	-2.34468800	0.39009900	-1.41047500
14	C	-2.19881300	-0.51164100	-0.19872900
15	C	-3.43293800	-0.87950800	0.54936300
16	C	-4.65790100	-0.49232500	0.15489600
17	C	-3.30832800	-1.72513000	1.79986700

18	H	0.33345300	-0.07447500	-1.20425500
19	C	0.17984200	-3.06897400	-1.48798100
20	O	2.21515100	-1.78376600	-1.58640700
21	H	0.65984800	0.47797700	1.43527500
22	O	3.63701100	-0.08695400	-0.00560400
23	C	-1.49313700	2.75814900	1.13153200
24	C	3.04972400	1.40159400	1.71927900
25	H	-0.99824500	-1.60602300	1.10242300
26	H	2.61891900	-3.06088000	0.39639500
27	H	0.99842200	-3.42281500	0.96673600
28	H	1.01527100	-1.60882300	2.41798400
29	H	2.70085100	-1.33988500	2.01327100
30	H	2.10493500	1.29169400	-1.60630100
31	H	3.29252700	2.26987200	-0.78571400
32	H	1.21652400	3.20750800	0.61114500
33	H	1.60905900	3.80048700	-0.98257900
34	H	-0.10184000	2.29930400	-2.02782100
35	H	-3.38815900	2.06966200	-0.49548200
36	H	-2.60591900	2.44043600	-2.02171300
37	H	-3.21255500	0.08057600	-2.00000200
38	H	-1.48226100	0.26357600	-2.06585000
39	H	-5.53734800	-0.77956200	0.72022700
40	H	-4.83319500	0.11082800	-0.72592400
41	H	-2.85907700	-2.69968600	1.58542400
42	H	-2.67943100	-1.24117600	2.55335800
43	H	-4.29062600	-1.89874700	2.24087200
44	H	0.76196700	-3.95735600	-1.74569500
45	H	-0.20310300	-2.62848800	-2.41442800
46	H	-0.67622400	-3.37648500	-0.88568100
47	H	1.88895200	-1.45524800	-2.43327100
48	H	3.29293800	-0.67007600	-0.70640500
49	H	-0.59344800	3.10802000	1.63846500
50	H	-2.26166200	3.53304400	1.23830600
51	H	-1.86527600	1.87570100	1.66329100
52	H	3.27992700	0.66017500	2.48658300
53	H	3.96809200	1.95105400	1.49714900
54	H	2.31998100	2.10030000	2.13239000

B3LYP/6-311G(d,p) Energy =-932.05410311 a.u.; Population = 8.06%

Compound **2**<br>Conformer 14		Standard orientation<br>(Ångstroms)		
I	atom	X	Y	Z
1	C	0.37594500	-0.73182200	-0.34900000

2	C	1.28341900	-0.15649800	0.79685400
3	C	2.52335700	0.72449800	0.42472300
4	C	-1.00450700	-0.96706500	0.21514700
5	C	1.06830800	-2.07538400	-0.76283700
6	C	1.62499100	-2.62831700	0.57924100
7	C	1.68204100	-1.44487800	1.58170400
8	C	2.31020000	1.79334600	-0.67003900
9	C	1.25709200	2.90363500	-0.42796700
10	C	-0.10480300	2.54801700	-0.95231500
11	C	-1.27207500	2.44698400	-0.30923100
12	C	-2.48302700	1.91177400	-1.06194500
13	C	-2.34894000	0.40475300	-1.40738200
14	C	-2.20288900	-0.50561600	-0.20202900
15	C	-3.43705300	-0.88018400	0.54216600
16	C	-4.66237100	-0.48894000	0.15213800
17	C	-3.31322300	-1.73815300	1.78448300
18	H	0.33522700	-0.07849000	-1.21624100
19	C	0.16280600	-3.06164700	-1.49273700
20	O	2.12681300	-1.79889600	-1.69276800
21	H	0.66284700	0.48295000	1.42909400
22	O	3.58739600	-0.09802400	-0.15020100
23	C	-1.49035000	2.76476600	1.14682300
24	C	3.08135900	1.38267800	1.69600900
25	H	-1.00406300	-1.60804500	1.09117500
26	H	2.60591700	-3.07421800	0.40030800
27	H	0.98116000	-3.42412300	0.96190400
28	H	0.97105700	-1.60647400	2.39431600
29	H	2.65874000	-1.36396600	2.06241800
30	H	2.09251400	1.28390900	-1.61356000
31	H	3.28970200	2.26121600	-0.80192600
32	H	1.22485600	3.18390700	0.62642100
33	H	1.61135600	3.79429600	-0.96136700
34	H	-0.10520200	2.29555100	-2.01390500
35	H	-3.38948000	2.07600500	-0.47402800
36	H	-2.61417700	2.46096700	-2.00027400
37	H	-3.21697200	0.10041900	-1.99941500
38	H	-1.48592900	0.28253200	-2.06272800
39	H	-5.54166300	-0.78178600	0.71487400
40	H	-4.83788000	0.12295800	-0.72261400
41	H	-2.86333300	-2.71034900	1.56090300
42	H	-2.68512200	-1.26144400	2.54328700
43	H	-4.29582000	-1.91638800	2.22304600
44	H	0.74044000	-3.94951800	-1.76408100

45	H	-0.22328300	-2.61354400	-2.41233500
46	H	-0.68277000	-3.37148200	-0.87743800
47	H	2.77125200	-1.22033100	-1.24434900
48	H	4.01699100	-0.58942800	0.55935100
49	H	-0.58919600	3.11199100	1.65314600
50	H	-2.25586600	3.54249100	1.25402500
51	H	-1.86542900	1.88383200	1.67905300
52	H	3.34686300	0.63719300	2.44988200
53	H	3.97940000	1.95565500	1.45365800
54	H	2.35251800	2.05737800	2.14832400

B3LYP/6-311G(d,p) Energy =-932.05302675 a.u.; Population = 2.57%

Compound **2**<br>Conformer 15		Standard orientation<br>(Ångstroms)		
I	atom	X	Y	Z
1	C	0.41378000	-0.79876100	-0.29241900
2	C	1.32561700	-0.12632500	0.79468900
3	C	2.52545100	0.76795300	0.35092000
4	C	-0.95850700	-0.99430400	0.30843900
5	C	1.11116000	-2.16302400	-0.60757300
6	C	1.66899900	-2.60900800	0.77041800
7	C	1.78582800	-1.34293700	1.66039400
8	C	2.26469000	1.76092900	-0.80821300
9	C	1.20035500	2.87330200	-0.62372400
10	C	-0.16661300	2.45467000	-1.08493600
11	C	-1.32320200	2.41188700	-0.41638700
12	C	-2.54198700	1.79449000	-1.09304400
13	C	-2.35708700	0.27964700	-1.36866100
14	C	-2.15333400	-0.53711300	-0.10761900
15	C	-3.37216300	-0.82501700	0.71087200
16	C	-3.37910000	-0.68139700	2.04282800
17	C	-4.61509900	-1.29055300	-0.01742200
18	H	0.35494400	-0.21434000	-1.20753600
19	C	0.20903800	-3.20531600	-1.26010200
20	O	2.16859300	-1.95357400	-1.55715400
21	H	0.69695700	0.53698600	1.39212900
22	O	3.57009600	-0.13824000	-0.11371200
23	C	-1.52144100	2.87957300	1.00143900
24	C	3.07726300	1.51824100	1.57420500
25	H	-0.96767600	-1.59034700	1.21906700
26	H	2.63076600	-3.10521000	0.62350700
27	H	0.99850700	-3.33998200	1.22987800
28	H	1.13959600	-1.43439800	2.53592300

29	H	2.80112000	-1.21093900	2.03012400
30	H	2.03755500	1.18557100	-1.71032200
31	H	3.22963300	2.24665900	-0.99721900
32	H	1.18997400	3.23114400	0.40717800
33	H	1.52592200	3.72628700	-1.23211600
34	H	-0.18092500	2.09718000	-2.11537000
35	H	-3.43035200	1.94730500	-0.47279500
36	H	-2.73940900	2.29419500	-2.04739400
37	H	-3.23571900	-0.08595300	-1.90864500
38	H	-1.51057200	0.14761100	-2.04342000
39	H	-4.25536800	-0.93188000	2.63206200
40	H	-2.51498700	-0.30839900	2.58020500
41	H	-5.04148900	-0.49617900	-0.63803800
42	H	-4.39271400	-2.12975500	-0.68455800
43	H	-5.38313100	-1.60593900	0.69086300
44	H	0.78909400	-4.11006800	-1.46182100
45	H	-0.17774800	-2.83120800	-2.21212400
46	H	-0.63609000	-3.46911900	-0.62315000
47	H	2.80867200	-1.33814800	-1.15492300
48	H	4.31286000	0.38845200	-0.43218600
49	H	-0.60815200	3.25626400	1.46293600
50	H	-2.26893900	3.68149600	1.03430400
51	H	-1.91200000	2.06716000	1.62319200
52	H	3.36012800	0.82178100	2.36441000
53	H	3.96571400	2.09235600	1.29444000
54	H	2.34242200	2.21429200	1.98369400

B3LYP/6-311G(d,p) Energy =-932.05148514 a.u.; Population = 0.50%

Compound **2**<br>Conformer 16		Standard orientation<br>(Ångstroms)		
I	atom	X	Y	Z
1	C	-0.35300400	-0.72286600	0.34886100
2	C	-1.39092600	-0.48029000	-0.81191100
3	C	-2.50174300	0.59438800	-0.61754000
4	C	0.98762500	-1.01292600	-0.28012700
5	C	-0.90976200	-1.96664100	1.12202400
6	C	-1.44450600	-2.85933300	-0.01615600
7	C	-1.99642300	-1.90109400	-1.09676600
8	C	-2.05147800	1.95630300	-0.04006700
9	C	-1.05055700	2.83113700	-0.83566600
10	C	0.39559800	2.42189100	-0.68997100
11	C	1.16072800	2.62244100	0.39063800
12	C	2.53176000	1.99081000	0.50044400

13	C	2.44751000	0.53849400	1.06517400
14	C	2.20760200	-0.50554200	-0.00748500
15	C	3.38500400	-0.95388800	-0.80270500
16	C	4.62891200	-0.49825300	-0.57476400
17	C	3.17906500	-1.96046000	-1.91621900
18	H	-0.29051000	0.11655600	1.03786100
19	C	0.09808600	-2.66809900	2.02544300
20	O	-1.96775100	-1.55491000	1.99921500
21	H	-0.83170900	-0.14338800	-1.68678700
22	O	-3.44438500	0.03748300	0.34503900
23	C	0.71033900	3.37691100	1.61757700
24	C	-3.25141700	0.79870300	-1.94383100
25	H	0.92902000	-1.74751400	-1.07748200
26	H	-2.20181600	-3.54849100	0.36463200
27	H	-0.62699500	-3.46408600	-0.41889800
28	H	-1.73717200	-2.24459500	-2.09999100
29	H	-3.08339600	-1.86086800	-1.04541900
30	H	-1.66427100	1.79029600	0.96771200
31	H	-2.97283100	2.54100200	0.08081200
32	H	-1.32697200	2.84816100	-1.89313500
33	H	-1.18111700	3.85885500	-0.48242400
34	H	0.82602700	1.85901800	-1.51436700
35	H	3.02499700	1.96789300	-0.47402000
36	H	3.16255700	2.59255800	1.16294800
37	H	3.37244000	0.30379700	1.59720600
38	H	1.65671100	0.49363100	1.81581000
39	H	5.46263300	-0.84649700	-1.17418700
40	H	4.86545700	0.22286200	0.19620700
41	H	4.12784700	-2.18512300	-2.40489400
42	H	2.76300300	-2.90036600	-1.54068000
43	H	2.48750500	-1.58502900	-2.67669400
44	H	-0.39584500	-3.49261200	2.54704000
45	H	0.48110000	-1.97365200	2.77804600
46	H	0.94178900	-3.06788600	1.46180600
47	H	-2.59270700	-1.00179200	1.49581300
48	H	-4.08824200	0.71633400	0.57810200
49	H	-0.28485300	3.80982400	1.51829900
50	H	0.70379800	2.72364800	2.49850700
51	H	1.41327700	4.18764500	1.84077000
52	H	-3.66617000	-0.14275200	-2.30634400
53	H	-4.07800100	1.50186900	-1.80470100
54	H	-2.59293800	1.20001400	-2.71730000

B3LYP/6-311G(d,p) Energy =-932.05466976 a.u.; Population = 14.70%

Compound **2**<br>Conformer 17		Standard orientation<br>(Ångstroms)		
I	atom	X	Y	Z
1	C	0.39772100	-0.77960000	-0.29732700
2	C	1.45109900	-0.41494800	0.81869800
3	C	2.51112000	0.68953700	0.50781800
4	C	-0.93037200	-1.02996400	0.37555000
5	C	0.96583800	-2.07689700	-0.96205100
6	C	1.52339400	-2.85339900	0.24665100
7	C	2.11773900	-1.78607500	1.18976400
8	C	1.97399100	2.00527700	-0.09754300
9	C	0.95395700	2.85222600	0.70384200
10	C	-0.48313400	2.40499500	0.57534800
11	C	-1.25424200	2.55207300	-0.50968200
12	C	-2.60558000	1.87512100	-0.60257300
13	C	-2.46043400	0.39163200	-1.06036300
14	C	-2.15000300	-0.54030500	0.09339300
15	C	-3.29904500	-0.89759700	0.98308800
16	C	-3.19894600	-0.83259600	2.31724100
17	C	-4.59167200	-1.33474300	0.32773600
18	H	0.30999600	-0.00453600	-1.05728300
19	C	-0.03509900	-2.87385000	-1.79016900
20	O	2.01573800	-1.73070100	-1.87776600
21	H	0.89825100	-0.04047900	1.68315100
22	O	3.43016500	0.21547500	-0.52851900
23	C	-0.82372100	3.27816900	-1.76094400
24	C	3.32657800	0.99008200	1.77339000
25	H	-0.87355400	-1.70809900	1.22467200
26	H	2.26073300	-3.59195100	-0.07605400
27	H	0.71083900	-3.39471100	0.74017100
28	H	1.95114600	-2.03830600	2.23846100
29	H	3.19937900	-1.73975300	1.05501000
30	H	1.56237000	1.78603100	-1.08552500
31	H	2.86444000	2.61687400	-0.27292700
32	H	1.23789900	2.88472200	1.75925200
33	H	1.05234000	3.88178700	0.34493400
34	H	-0.89948300	1.86004500	1.41831100
35	H	-3.12300100	1.90142600	0.36066300
36	H	-3.23845200	2.40766600	-1.31923100
37	H	-3.39236900	0.07577700	-1.53655300
38	H	-1.68860300	0.32359600	-1.82947300

39	H	-4.02165400	-1.12910600	2.96011100
40	H	-2.29668700	-0.47949400	2.80321100
41	H	-5.29578200	-1.70950700	1.07266800
42	H	-5.07630700	-0.50793700	-0.20101200
43	H	-4.41485700	-2.12363900	-0.41056900
44	H	0.47143900	-3.73064100	-2.24324900
45	H	-0.43935400	-2.25560100	-2.59625700
46	H	-0.86431500	-3.24057400	-1.18400000
47	H	2.58870000	-1.06203900	-1.45947200
48	H	4.19958800	-0.18169500	-0.10663500
49	H	0.15473000	3.74978700	-1.67164200
50	H	-0.78810800	2.59489900	-2.61822400
51	H	-1.55353000	4.05490800	-2.01643500
52	H	3.81381000	0.09189400	2.16094700
53	H	4.09847600	1.73231400	1.55448600
54	H	2.68956400	1.38332300	2.56757600

B3LYP/6-311G(d,p) Energy =-932.05141434 a.u.; Population = 0.47%

Compound **2**<br>Conformer 18		Standard orientation<br>(Ångstroms)		
I	atom	X	Y	Z
1	C	-0.28279500	-0.70097000	0.26978400
2	C	-1.30326900	-0.48962100	-0.90458300
3	C	-2.54683400	0.43205000	-0.67512200
4	C	1.07380500	-0.98173400	-0.33120000
5	C	-0.84689500	-1.92551800	1.06719900
6	C	-1.44110500	-2.83887300	-0.03733500
7	C	-1.69792400	-1.95202600	-1.28427900
8	C	-2.27744100	1.75358200	0.07538400
9	C	-1.36552600	2.81671000	-0.58209700
10	C	0.11191300	2.50680100	-0.56062700
11	C	0.92564600	2.61922200	0.49629600
12	C	2.35451600	2.12818800	0.43399100
13	C	2.51032100	0.66961900	0.96797900
14	C	2.26882300	-0.42873900	-0.05748600
15	C	3.48103000	-0.93025100	-0.77896600
16	C	3.74071400	-2.24048400	-0.87515300
17	C	4.42385400	0.08624100	-1.38480500
18	H	-0.23146400	0.15252400	0.94049400
19	C	0.16994700	-2.63441900	1.95513200
20	O	-1.86631700	-1.47286300	1.97285400
21	H	-0.76537800	-0.03241900	-1.73791900
22	O	-3.52153400	-0.22632700	0.19508300

23	C	0.49429100	3.15239900	1.84064300
24	C	-3.23409500	0.71315800	-2.01885700
25	H	1.07174900	-1.74578100	-1.10628500
26	H	-2.35136000	-3.31087600	0.33890300
27	H	-0.74177800	-3.64297700	-0.27948000
28	H	-1.08186300	-2.29011700	-2.11969200
29	H	-2.72908800	-2.03122700	-1.63331900
30	H	-1.89733900	1.51295500	1.07148600
31	H	-3.26374100	2.20321600	0.22692400
32	H	-1.68789300	2.99408000	-1.61178800
33	H	-1.55317500	3.75571700	-0.05212700
34	H	0.53590900	2.10613500	-1.47837400
35	H	2.72651600	2.18415400	-0.59064600
36	H	2.99104900	2.78262700	1.03989300
37	H	3.52323000	0.55103200	1.36384700
38	H	1.84111800	0.52987600	1.82039900
39	H	4.61168600	-2.60378500	-1.41131800
40	H	3.09837100	-2.98400800	-0.41773500
41	H	5.27322800	-0.40675200	-1.86101900
42	H	3.91348300	0.69562800	-2.13802000
43	H	4.81505000	0.77681400	-0.63155000
44	H	-0.32472100	-3.44724700	2.49407400
45	H	0.57995500	-1.93900100	2.69253300
46	H	0.99500500	-3.05151800	1.37675600
47	H	-2.56411600	-1.04081400	1.44612200
48	H	-3.99994500	-0.88677500	-0.31910300
49	H	-0.54485700	3.47918200	1.85986900
50	H	0.61856500	2.39539500	2.62381000
51	H	1.12395200	4.00136500	2.13100700
52	H	-3.54636800	-0.21252600	-2.50911900
53	H	-4.12152100	1.33120900	-1.86223800
54	H	-2.56531000	1.23543300	-2.70527900

B3LYP/6-311G(d,p) Energy =-932.04997889 a.u.; Population = 0.10%

Compound **2**<br>Conformer 19		Standard orientation<br>(Ångstroms)		
I	atom	X	Y	Z
1	C	0.43582800	-0.79270400	-0.29054700
2	C	1.34817400	-0.11061700	0.79267500
3	C	2.53994200	0.80796900	0.34077200
4	C	-0.93617100	-0.99867900	0.31079300
5	C	1.11756300	-2.15664800	-0.57898300
6	C	1.64103500	-2.60369400	0.80273400

7	C	1.85085800	-1.31923600	1.64786500
8	C	2.23955600	1.80786300	-0.80913600
9	C	1.15047600	2.89632400	-0.62391600
10	C	-0.20728800	2.45568700	-1.09089700
11	C	-1.36717900	2.39616000	-0.42929400
12	C	-2.57096900	1.75427300	-1.10977200
13	C	-2.35655100	0.24123000	-1.37428300
14	C	-2.13654000	-0.55893400	-0.10616000
15	C	-3.34768100	-0.85033300	0.72298600
16	C	-3.34796600	-0.69179700	2.05312000
17	C	-4.58931500	-1.33656000	0.00648800
18	H	0.37549400	-0.21685300	-1.21146000
19	C	0.24777400	-3.19453500	-1.28198600
20	O	2.23004500	-1.83808900	-1.46717400
21	H	0.70469100	0.53179800	1.39785100
22	O	3.66387300	0.02314600	-0.09818900
23	C	-1.58108400	2.86685900	0.98547700
24	C	3.05762400	1.57310500	1.56749700
25	H	-0.93524200	-1.58142400	1.22984500
26	H	2.55904100	-3.18667100	0.68962100
27	H	0.90733600	-3.26319200	1.27322500
28	H	1.29130800	-1.38640600	2.58319200
29	H	2.89989000	-1.19332800	1.90749100
30	H	2.02355200	1.23402200	-1.71668300
31	H	3.19739200	2.30324800	-0.99403600
32	H	1.12802500	3.24924100	0.40882600
33	H	1.45938800	3.76019200	-1.22666300
34	H	-0.21098900	2.09388900	-2.12025300
35	H	-3.46601000	1.89489200	-0.49623100
36	H	-2.77149300	2.24353500	-2.06882700
37	H	-3.22795700	-0.14737500	-1.91000900
38	H	-1.50765100	0.12087100	-2.04825500
39	H	-4.21837000	-0.94384000	2.65024200
40	H	-2.48468800	-0.30395400	2.58119900
41	H	-5.02704500	-0.55350200	-0.62058500
42	H	-4.36185700	-2.18088900	-0.65238800
43	H	-5.35016200	-1.65179200	0.72249100
44	H	0.82535900	-4.10663800	-1.46569600
45	H	-0.10099300	-2.81191600	-2.24437300
46	H	-0.62104500	-3.46397500	-0.67983500
47	H	2.70784400	-2.65664100	-1.64962600
48	H	3.31025700	-0.64068100	-0.71806600
49	H	-0.67514600	3.25711200	1.45004500

50	H	-2.33900100	3.65936600	1.01066400
51	H	-1.96466300	2.05240200	1.60885800
52	H	3.34262300	0.88354600	2.36429500
53	H	3.94277200	2.15121800	1.28963200
54	H	2.30748300	2.25889600	1.96583000

B3LYP/6-311G(d,p) Energy =-932.05168813 a.u.; Population = 0.62%

Compound **2**<br>Conformer 20		Standard orientation<br>(Ångstroms)		
I	atom	X	Y	Z
1	C	0.37166600	-0.74477700	-0.29134200
2	C	1.29979600	-0.15384300	0.82689900
3	C	2.54783400	0.69700400	0.42915100
4	C	-1.01007000	-0.94765900	0.28697400
5	C	1.03734900	-2.11265000	-0.66846300
6	C	1.57455600	-2.63828300	0.69079300
7	C	1.69200700	-1.42238400	1.64810400
8	C	2.36274300	1.71486200	-0.72216600
9	C	1.34186900	2.86806500	-0.55012600
10	C	-0.03218800	2.51308600	-1.04142700
11	C	-1.18735200	2.44759500	-0.37252300
12	C	-2.42584300	1.92394400	-1.08480400
13	C	-2.38354400	0.39446600	-1.36090700
14	C	-2.20514400	-0.50586100	-0.14808800
15	C	-3.44775700	-0.94662900	0.56056100
16	C	-3.62767100	-2.22526800	0.91901600
17	C	-4.52623900	0.07758700	0.83862600
18	H	0.32886000	-0.11783000	-1.17829200
19	C	0.11706500	-3.09872100	-1.37992400
20	O	2.10686300	-1.88263600	-1.59937200
21	H	0.69544700	0.51464000	1.44344000
22	O	3.56880600	-0.24411000	-0.02129700
23	C	-1.36804700	2.81046500	1.07850100
24	C	3.09572400	1.40336400	1.67968700
25	H	-1.02992300	-1.55784100	1.18775800
26	H	2.53414700	-3.13366100	0.52794400
27	H	0.89362200	-3.38778900	1.10260100
28	H	1.00631800	-1.53730600	2.49027700
29	H	2.69372100	-1.33968800	2.06626600
30	H	2.13787000	1.16108500	-1.63811000
31	H	3.35222000	2.16186900	-0.87695300
32	H	1.32640600	3.21714400	0.48354400
33	H	1.71325800	3.71162000	-1.14465100

34	H	-0.05596300	2.22919800	-2.09478300
35	H	-3.31792000	2.17491700	-0.50720600
36	H	-2.53616900	2.42295800	-2.05331300
37	H	-3.30748900	0.11756500	-1.88072400
38	H	-1.57198600	0.20448900	-2.06613500
39	H	-4.52194600	-2.54035200	1.44696600
40	H	-2.89712200	-2.99216500	0.69044600
41	H	-5.36562100	-0.37858400	1.36630100
42	H	-4.14549300	0.90325800	1.44766700
43	H	-4.91199000	0.51683900	-0.08643900
44	H	0.67964000	-4.00335400	-1.62652200
45	H	-0.25552600	-2.66611100	-2.31256200
46	H	-0.73762300	-3.37860800	-0.76343800
47	H	2.77385800	-1.33066100	-1.15120300
48	H	4.34349500	0.25798100	-0.30129700
49	H	-0.45468700	3.17378000	1.55008200
50	H	-2.13214600	3.58985100	1.18229400
51	H	-1.72450600	1.94573500	1.64852200
52	H	3.31877900	0.68286100	2.46780600
53	H	4.01975200	1.93703500	1.43727900
54	H	2.38285100	2.12891800	2.07653200

B3LYP/6-311G(d,p) Energy =-932.04924618 a.u.; Population = 0.05%
